# Closed‐Loop Decoding and Intervention of Pain: A Novel BMI Strategy Integrating *θ*‐Band Detection and Mechano‐Electro‐Biological Coupled Hydrogels

**DOI:** 10.1002/advs.76269

**Published:** 2026-06-26

**Authors:** Yun Ji, Tao Li, Guoqiang Lei, Huichun Luo, Xiuqian Guo, Jiao Xiang, Tao Shi, Weitang Liu, Yuxin Zhang, Xiaoyu Liao, Shutao Zhao, Jiayun Wu, Wangao Zhang, Wenhui Liu, Chuanglong He, Shuo Chen, Tao Wu, Ke Ma

**Affiliations:** ^1^ Department of Pain Medicine Xinhua Hospital Affiliated to Shanghai Jiao Tong University Shanghai China; ^2^ Department of Orthopedics School of Medicine Xin Hua Hospital Affiliated to Shanghai Jiao Tong University Shanghai China; ^3^ State Key Laboratory of Advanced Fiber Materials College of Biological Science and Medical Engineering Donghua University Shanghai China; ^4^ Department of Anesthesiology Renji Hospital Affiliated to Shanghai Jiao Tong University Key Laboratory of Anesthesiology (Shanghai Jiao Tong University) Ministry of Education Shanghai China; ^5^ Department of Pain The Second Affiliated Hospital of Anhui Medical University Hefei China; ^6^ Department of Pain The Second Affiliated Hospital of Kunming Medical University Kunming China; ^7^ Institute of Infectious Disease and Biosecurity Fudan University Shanghai China; ^8^ Department of Oral Surgery Shanghai Ninth People's Hospital Affiliated to Shanghai Jiao Tong University Shanghai China; ^9^ Department of Rehabilitation Medicine The Affiliated Suzhou Hospital of Nanjing Medical University Suzhou Municipal Hospital Suzhou China; ^10^ Institute for Developmental and Regenerative Cardiovascular Medicine MOE‐Shanghai Key Laboratory of Children's Environmental Health Xin Hua Hospital Affiliated to Shanghai Jiao Tong University Shanghai China; ^11^ Shanghai Jiao Tong University School of Medicine Shanghai China; ^12^ Department of Pain Medicine First Affiliated Hospital of Anhui University of Chinese Medicine Hefei China; ^13^ People's Hospital Affiliated to Fujian University of Traditional Chinese Medicine Fuzhou China; ^14^ School of Health Science and Engineering University of Shanghai for Science and Technology Shanghai China; ^15^ Centre for Collaborative Research Shanghai University of Medicine and Health Sciences Shanghai China; ^16^ Department of Pain Medicine Ninth People's Hospital Affiliated to Shanghai Jiao Tong University School of Medicine Shanghai China

**Keywords:** biocompatibility, biomedical engineering, electrocorticography, electroencephalography, inflammation, medicine, neuropathic pain, stimulation

## Abstract

Neuropathic pain (NP) lacks objective biomarkers and effective therapies. We developed a closed‐loop brain‐machine interface (BMI) using a novel hydrogel electrode for integrated NP management. Clinically, we identified enhanced prefrontal θ‐band (4–8 Hz) activity as a specific NP biomarker. To achieve stable long‐term recording, we engineered a PFAPT hydrogel with superior biocompatibility, anti‐swelling, and low impedance. In rats, it enabled stable electrocorticography (ECoG) acquisition for 28 d, outperforming screw electrodes, and validated θ‐band alterations in NP. Incorporated into a closed‐loop BMI, the hydrogel delivered θ‐triggered cortical stimulation, which increased pain thresholds and alleviated anxiety‐ and depression‐like behaviors in NP rats. Spatial transcriptomics revealed that stimulation bidirectionally regulated inflammation, central sensitization, and affective pathways. This work presents a hydrogel‐based closed‐loop BMI as a precise diagnostic and therapeutic platform for NP.

## Graphical abstract

A closed‐loop brain–machine interface with a PFAPT hydrogel electrode identifies prefrontal θ‐band as a neuropathic pain biomarker. The hydrogel enables stable 28 d ECoG recording and delivers θ‐triggered cortical stimulation, alleviating pain and affective behaviors in rats via bidirectional regulation of inflammation and central sensitization.

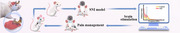

## 1 Introduction

Neuropathic pain (NP) refers to the pain caused by injury or disease affecting the somatosensory nervous system, including multiple sclerosis, spinal cord injuries, and herpes zoster, which is characterized by typical symptoms such as burning sensations, allodynia, numbness, and tingling [1, 2, 3]. With a global incidence rate of approximately 7% to 10%, NP has become a global health problem [1]. In clinical, pharmacotherapy remains the primary treatment modality yet it is frequently associated with adverse effects such as sedation, desensitization, and even addiction [4, 5, 6]. Non‐pharmacological intervention strategies, including brain‐machine interface (BMI), have shown promising potential in cognition and pain management [7, 8, 9]. However, there are still some shortcomings in current research, hindering further development. Firstly, self‐reported numerical rating scales are commonly used in clinical settings. Due to the individual subjectivity of pain ratings, the diagnosis and therapeutic effect of NP always lacks reliability and accuracy [10]. Secondly, the characteristic of NP is spontaneous pain attacks that are difficult to predict in terms of time and frequency, leading to poor efficacy of clinical intervention strategies [11]. Therefore, new breakthroughs are urgently needed to address these challenges. Closed‐loop BMI combines monitoring of neural signal dysfunction with therapeutic neural regulation by automatically analyzing neural activity (electroencephalograph (EEG), electrocorticography (ECoG), and local field potential (LFP) signals) detected by pain attacks and combining it with therapeutic brain stimulation to provide demand‐based analgesia [12, 13]. Very recently, closed‐loop BMI systems have achieved suppression of acute and chronic pain behavior [14]. Despite advances in signal processing and machine algorithms, the electrodes remain the most important part of BMI. Though great efforts have been made to improve the performance of BMI, there are still many challenges in constructing long‐term stable neural interfaces, achieving high‐quality signal acquisition, and ensuring effective neural regulation [15, 16].

Hydrogel electrodes show advantages in improving the signal‐to‐noise ratio (SNR) due to their biocompatibility and softness [17, 18, 19]. Despite the advancement of hydrogel electrodes in scientific research, their overall performance still falls short of clinical requirements, leading to ongoing struggles in further development. The ideal BMI electrode needs to integrate multiple properties for stable mechanical coupling and effective acquisition of physiological signals. For instance, it requires suitable mechanical compatibility for stable conformal contact with the brain in dynamic environments; stability in body fluids for consistent performance over extended periods; excellent tissue conformability to eliminate the interface impedance fluctuations; high conductivity and low interface impedance for precise electrical signal monitoring and stimulation; superior biocompatibility and immune regulatory properties to minimize immune reactions and glial scar formation. Although a few studies have reported soft and adhesive hydrogel electrodes, their application in NP management has been limited because of their poor anti‐swelling properties and low conductivity [20]. Excellent anti‐swelling property is crucial to prevent mechanical compression on neural tissues due to volume expansion and ensure consistent electrical recording stability during long‐term implantation. Generally, weakening the polymer‐water interaction and increasing the retraction force of the hydrogel molecular chain are considered effective strategies. The traditional anti‐swelling hydrogels rely on increased crosslinking density to enhance the elastic retraction force, which greatly compromises their softness, let alone the rigid units of conductive components [21]. Therefore, a sophisticated molecular design of hydrogel is required to simultaneously combine the abovementioned properties in one system.

**FIGURE 1:**
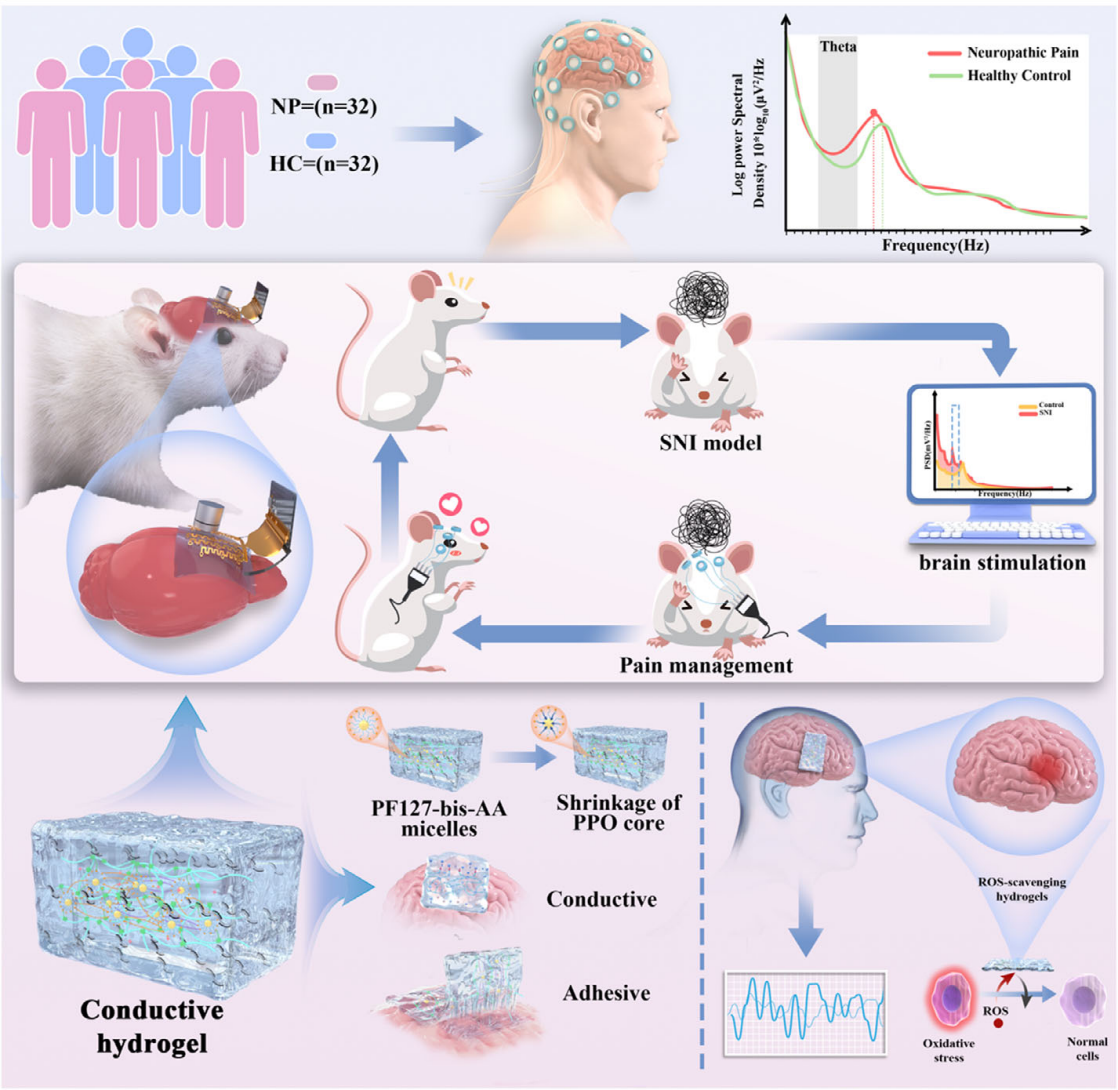
Schematic diagram of a closed‐loop BMI system for NP treatment.

Here, we propose a hydrogel‐based BMI system integrating diagnosis and therapeutics for NP (Figure 1). Initially, through a clinical cohort study, we systematically collected EEG signals from healthy subjects and patients with NP. Comprehensive analysis of their spectral characteristics revealed that significant alterations in low‐frequency θ band in the prefrontal cortex serve as a specific neuro‐biomarker for NP episodes. Then, we developed a closed‐loop BMI system based on a mechanical‐electrical‐biological coupling hydrogel electrode, composed of an alginate matrix, PF127 nano‐micelles, tannic acid (TA), and poly(3,4‐ethylenedioxythiophene): poly(styrenesulfonate) (PEDOT:PSS). Involving thermo‐sensitive crosslinks and adhesive moieties, the resultant hydrogel electrodes exhibit superior mechanical biocompatibility, excellent anti‐swelling, decent adhesion, low interface impedance, and anti‐inflammation. We further validated the potential of the hydrogel as an implantable electrode for acquiring ECoG signals and effectively managing NP. 28‐d signal acquisition comprehensively demonstrates that the hydrogel electrode outperformed commercial skull screw electrodes in long‐term signal acquisition. More importantly, the hydrogel‐based BMI system successfully captured the dynamic changes in θ band of NP model rats, further verified the clinical findings. Subsequently, the closed‐loop BMI system was successfully used to modulate and stimulate the brain functional regions of NP model rats via direct cortical electrical stimulation (DCS). Significant changes in θ bands of the rats’ brains were observed, along with key indicators of increased pain thresholds and improved emotional disturbances in NP model rats, further validating the effective management of NP. Molecular heterogeneity and cellular dynamic characteristics of each molecular layer of the cortex were systematically investigated through spatial transcriptomics technology, and revealed the regulatory effect of DCS intervention on chronic pain‐related pathways.

## 2 Result and discussion

### 2.1 Θ Band Activity as a Potential Neuro‐Biomarker for NP

**FIGURE 2:**
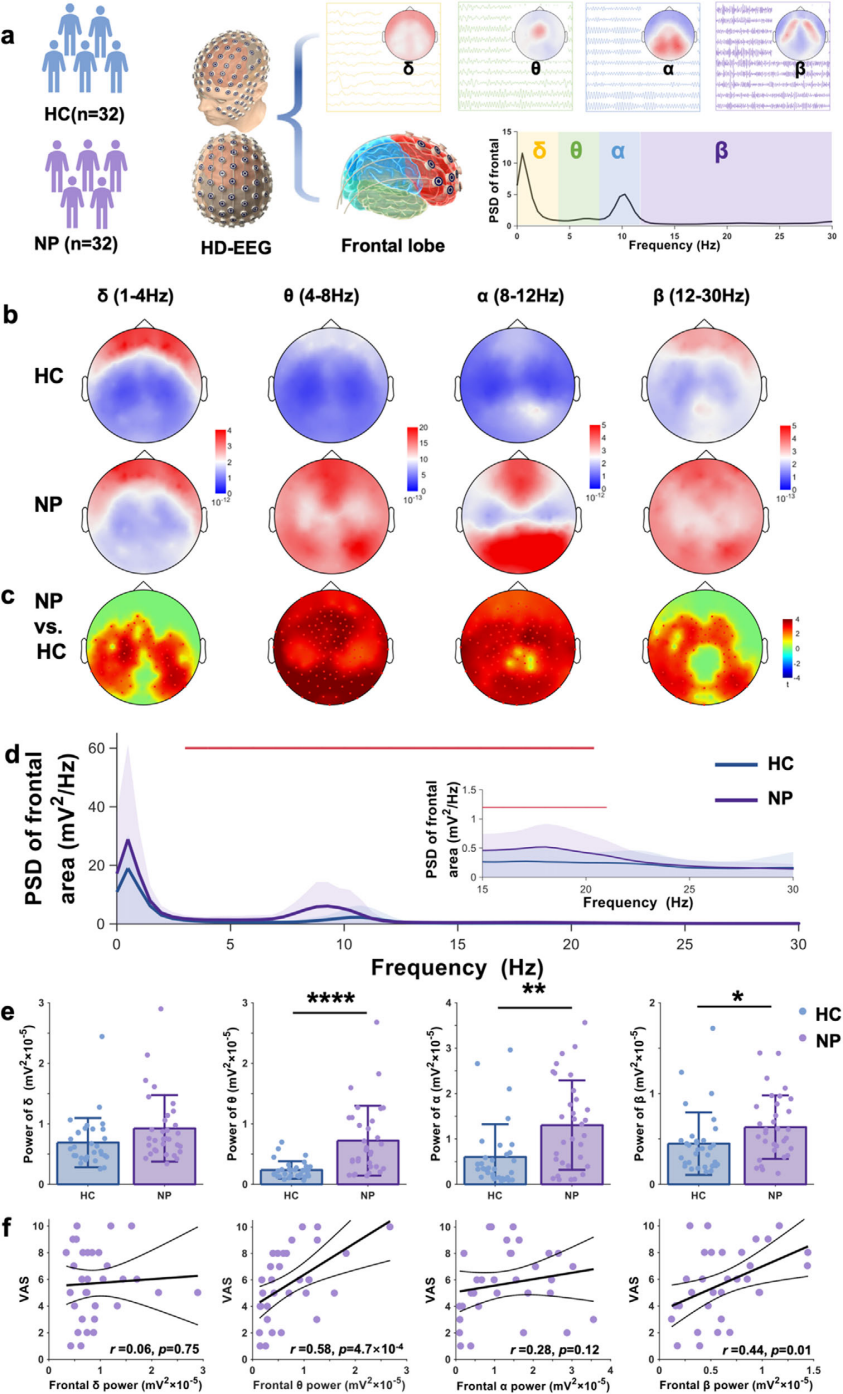
Abnormal neural oscillatory activity in NP. (a) Schematic diagram of clinical study. (b) The average oscillatory activity of HC and NP group. (c) The difference between NP and HC at each frequency band oscillation. (d) The PSD of frontal of NP and HC from 0 to 30 Hz. (e) The difference in oscillatory activities of the frontal lobe between NP and HC (from left to right: δ, θ, α, and β). (f) The relationships between oscillatory activities level of the frontal lobe and visual analog scale (VAS) score of NP patients.

Due to the individual subjectivity of traditional pain ratings, the diagnosis of NP always lacks reliability and accuracy [22]. Thus, we performed a clinical cohort study for measuring the EEG signal in the groups of NP and healthy controls (HC) patients by a 128‐channel high‐density EEG testing device (Figure 2a). A total of 32 NP and 32 HC were included in the study. Demographic variables were provided in Table S2. The two group were matched for age, sex, handedness, and educational years. At the sensor level, the activity level of NP was higher than HC in all frequency bands. As shown in Figure 2b,c, cluster‐based multiple comparison correction revealed that significantly increased δ activity in bilateral parietal lobes and part of occipital lobes, increased θ and α activity in the whole brain, increased β activity in bilateral occipital lobes and left of temporal lobes. Furthermore, through the overall evaluation of frontal lobe electrodes (Figure 2d,e), we found that the θ, α, and β activity levels in the frontal lobe of NP patients were significantly higher than those of HCs. The θ band in neuropathic pain patients shows significant abnormal enhancement, both across the whole brain and in key regions such as the frontal lobe, where its activity level is significantly higher than that of the HC group. This phenomenon indicates that changes in θ band activity occupy an important position in the neurobiological mechanisms of NP [23]. We further investigate the correlation between oscillatory activities level of the frontal lobe and visual analog scale (VAS) score of NP patients. As shown in Figure 2f, the activity level of *θ* (*r* = 0.58, *p* = 4.7 × 10^−4^) and β (*r* = 0.44, *p* = 0.01) oscillation in NP patients were significantly related to pain intensity. This demonstrates that θ band activity not only reflects abnormal changes in brain function during pain states but also provides an objective neurobiological marker for the subjective perception of NP [24]. This characteristic gives the θ band potential value in biomarker research and clinical applications.

### 2.2 Preparation and Characterization of PFAPT Hydrogels for BMI

**FIGURE 3:**
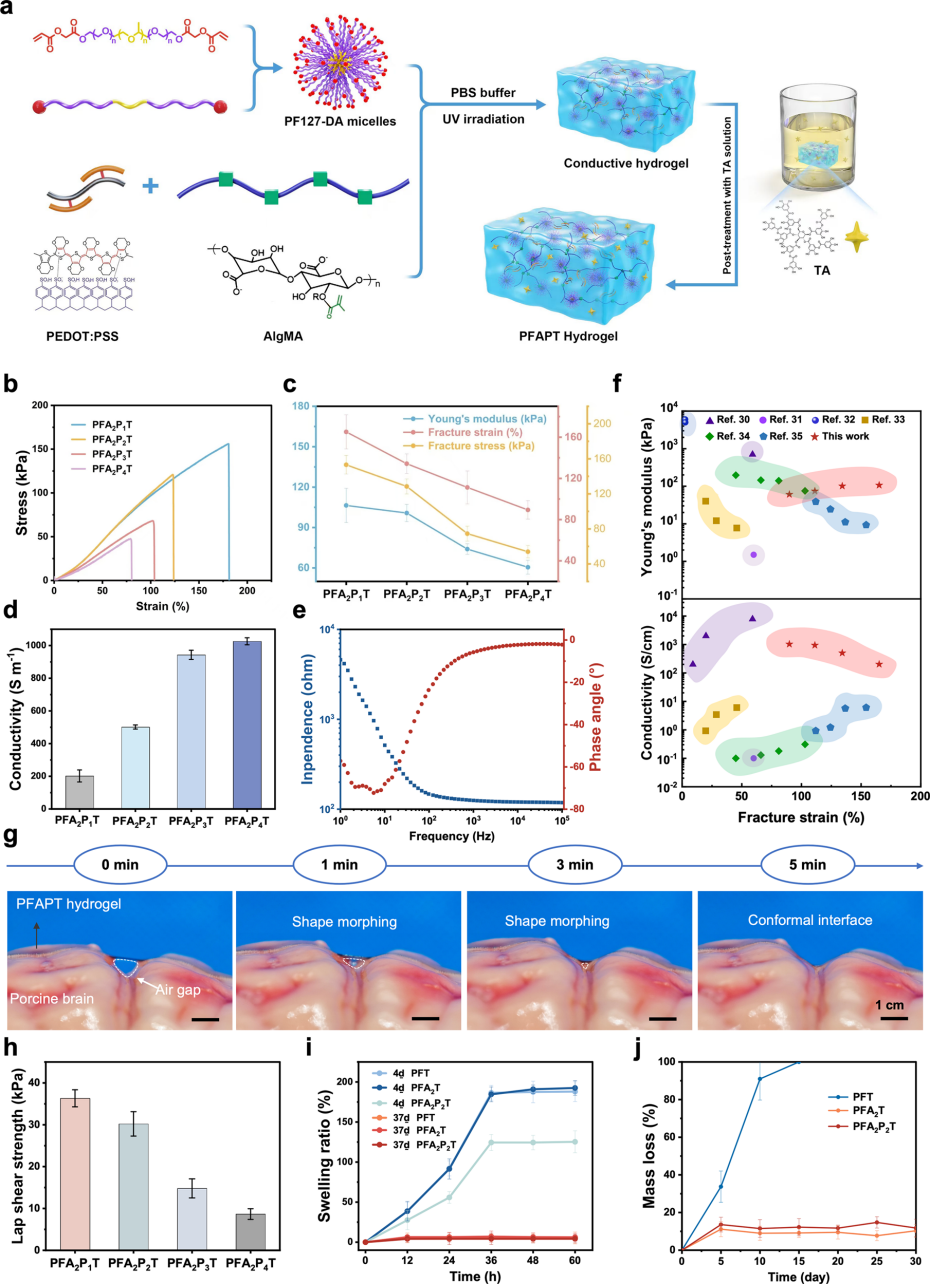
Preparation and characterization of PFAPT hydrogels. (a) Schematic diagram on the fabrication of PFAPT hydrogels. (b) Stress–strain curves of the PFA_2_P_1_T, PFA_2_P_2_T, PFA_2_P_3_T, and PFA_2_P_4_T hydrogels. (c) Young's modulus, fracture stress, and fracture strain of PFA_2_P_1_T, PFA_2_P_2_T, PFA_2_P_3_T, and PFA_2_P_4_T hydrogels. (d) Conductivity of the PFA2P1T, PFA2P2T, PFA2P3T, and PFA2P4T hydrogels. (e) Electrochemical impedance and phase angle of the PFAPT hydrogel. (f) Relationships of Young's modulus and conductivity versus fracture strain of a variety of PEDOT:PSS composites reported in references and this work. (g) PFAPT hydrogel mounted on porcine brain tissue illustrating its mechanical compatibility. (h) Lap shear strength of PFAPT hydrogels with different PEDOT:PSS loadings. (i) The variations of swelling ratios of PFT, PFA_2_T, and PFA_2_P_2_T hydrogels immersed in standard PBS buffer with different temperatures. (j) The mass loss of PFT, PFA_2_T, and PFA_2_P_2_T gels immersed in PBS buffer with different times.

To prepare mechanical‐electrical‐biological coupling hydrogels, pluronic F127 diacrylate (PF127‐DA) and alginate methacryloyl (AlgMA) were synthesized (Figures S1–S3). PF127 is an amphiphilic polymer consisting of polyethylene oxide (PEO) and polypropylene oxide (PPO) chains that are biocompatible and thermally sensitive to assembly into nanoscale micelles [25]. Sodium alginate is a natural polysaccharide that has the stability, solubility, viscosity, and safety required to prepare hydrogels [26]. PEDOT:PSS was used as a conductive filler in the hydrogels to achieve good conductivity. TA containing catechol groups not only enables the hydrogel adhesion ability, but also endow the hydrogel with anti‐inflammatory effects, reducing the level of pro‐inflammatory cytokines [27]. The hydrogels were firstly fabricated through photo‐crosslinking among PF127‐DA, AlgMA, and PEDOT:PSS by photo initiator lithium phenyl‐(2, 4, 6‐trimethylbenzoyl) phosphinate (LAP) within 30 s of UV illumination (365 nm, 100 mW cm–^2^). Then, the photo‐crosslinked hydrogels were soak in the TA solution to obtain the resultant hydrogel, named PFAPT hydrogels (Figure 3a). The synthesized PF127‐DA maintained its self‐assembly property, with transmission electron microscopy (TEM) images showing the formation of nano‐micelles with an average diameter of about 30 nm (Figure S4).

The mechanical and electrical properties of PFAPT hydrogels were systematically studied. The concentration of TA solution was determined as 5 mg mL^−1^ by in vitro cytocompatibility, which would be discussed in later section. The pure PF127‐DA gels were too soft and irreversible after deformation, so AlgMA was introduced to increase the density of crosslinking to improve the mechanical properties of the hydrogel (Figure 3b). The hydrogels were optimized by tuning the AlgMA and PEDOT:PSS contents. The detailed information is presented in Table S2. Stress–strain curves of PFT, PFA_1_T, PFA_2_T, and PFA_3_T were measured by tensile testing. PFT hydrogel of pure PF127‐DA was too soft and irreversible after deformation. With the increase of AlgMA, the mechanical performance of PFAPT hydrogels was improved. The stress of PFA_3_ was improved 4.8 times to 202.2 ± 12.7 kPa compared with pure PF127‐DA hydrogels (Figure S5), demonstrating that AlgMA doping provides an effective strategy to optimize the PFAPT hydrogel in a wide range. Considering the required low modulus of electrode in BMI applications, PFA_2_T was chosen for further experiments in this article. Further, we explored the effect of PEDOT:PSS doping on the mechanical and electrical properties of PFAPT hydrogels. As the mass concentration of PEDOT:PSS increases from 0.375% to 1.5%, the Young's modulus, fracture strain, and fracture stress of PFAPT hydrogels gradually decreased (Figure 3b,c). This mechanical compromise is primarily attributed to two factors. Structurally, the rigid PEDOT:PSS aggregates act as defects that disrupt the continuity of the covalently crosslinked PF127‐DA/AlgMA network, inducing stress concentration during deformation. Photochemically, excessive PEDOT:PSS impedes the photo‐polymerization efficiency via a UV‐shielding effect, thereby reducing the effective crosslinking density and overall structural integrity. The PFA_2_P_2_T hydrogels with a 0.75% mass concentration of PEDOT:PSS show a large range of reversible stretchability (140%) and the residual strain can hardly be observed even at a large tensile strain (Figure S6). Along with the regulating of mechanical properties of PFAPT hydrogels, the electrical conductivity also shows remarkable controllability ranging from 202 ± 36.9 to 1026.3 ± 21.4 S m^−1^ with the increase of PEDOT:PSS (Figure 3d). Given that the PF127‐DA and AlgMA networks are insulating, there is a trade‐off between mechanical flexibility and conductivity. Taking into account the compromise in mechanical flexibility and conductivity, PFA_2_P_2_T hydrogels presented suitable mechanical property (Young's modulus of 100.8 ± 6.4 kPa) and conductivity (501.6 ± 12.6 S m^−1^) meet the requirements of bioelectrode [28, 29]. To be a promising candidate for flexible brain electrodes, it needs to be able to establish an efficient electrical signaling pathway with brain tissues. Therefore, we further characterized the electrochemical properties of the PFA_2_P_2_T hydrogels. It exhibits low impedance (≈119.3 Ω) at 10^2–^10^5^ Hz (Figure 3e), indicating the low interference of the hydrogel during signal transduction. A phase value close to 0° indicates that a resistive charge transfer has occurred in the hydrogel. Cyclic voltammetry (CV) scans were performed on the Pt electrode for hydrogels. The results showed that the hydrogel had a CSC value of 14.65 mC cm^−2^, which was higher than that of the platinum electrode (0.34 mC cm^−2^) (Figure S7). As shown in Figure 3f, by comparing with hydrogels reported in other hydrogel‐based electrodes, PFAPT hydrogel achieves a balance among conductivity and modulus [30, 31, 32, 33, 34, 35]. These excellent mechanical and electrochemical properties, demonstrating the applicability of PFAPT hydrogels for bioelectronic applications.

The spontaneous shape‐morphing property of the PFAPT hydrogel film was investigated in vitro using porcine brain tissue. The PFAPT hydrogel film was autonomously shape‐morphed into the concave flexural of the cortex without any externally applied driving force, leading to complete conformation to surface profile, which demonstrate the excellent mechanical compatibility of our hydrogel (Figure 3g). Besides, we also investigated the lap shear strength of PFAPT hydrogels. The lap shear test was conducted by putting the PFAPT hydrogel films between two pieces of porcine skin (Figure S8). We observe that the shear adhesion force decreases from 36.3 ± 2.0 to 8.7 ± 1.3 kPa as the mass concentration of PEDOT:PSS increased as shown in Figure 3h. The decent lap shear adhesion strength is significant to improve the stability and durability of PFAPT hydrogel electrodes. Before the in vivo application of the PFAPT hydrogel, its on‐brain adhesion was investigated on the in vitro porcine cortex (Figure S9). The PFAPT hydrogel electrode was conformally mounted on the uneven wrinkled cortex surface and spontaneously shape‐morphed into the various valleys with different depths. Such tissue‐adhesion performance was stably maintained when shear stress was applied to the PFAPT hydrogel films. Considering the successful in vitro demonstration, our PFAPT hydrogel electrode is highly suitable for achieving the ultimate brain‐device interface.

Superior anti‐swelling property is essential for the hydrogel‐based BMI applications. The swelling behavior of the hydrogel was investigated in vitro. As a swelling control, PFT, PFA_2_T, and PFA_2_P_1_T hydrogels immersed at 4°C have swelling behavior obviously in 12 h, and reached the swelling equilibrium in 36 h with a swelling ratio of more than 100%. However, PFT, PFA_2_T, and PFA_2_P_1_T hydrogels immersed at 37°C reached swelling ratio of 2.58 ± 1.49% after 12 h (Figure 3i). The photograph in Figure S10 visually illustrates the swelling behavior of hydrogels at different temperatures, demonstrating the excellent anti‐swelling properties of PFAPT hydrogels at human body temperature. Therefore, temperatures contributed significantly to the anti‐swelling of PFAPT hydrogels, this is down to thermally sensitive to assembly into nanoscale micelles. When exposed to body temperature, the hydrophobic interactions between PPO chains become stronger, leading to the shrinkage of PPO cores and counterbalancing the extension of hydrophilic PEG chains, which results in the non‐expansion of the hydrogel volume macroscopically [36]. The stability for the long‐term applications of PFAPT hydrogels was studied by testing their degradation behavior in vitro. Pure PF127‐DA hydrogels completely degraded within 15 d in the PBS solutions. The introduction of AlgMA components into the hydrogel reduced the degradation rate of the PFAPT hydrogel (Figure 2f), which may be achieved by increasing the cross‐linking density. PFA_2_T and PFA_2_P_2_T hydrogels showed no significant degradation behavior during 30 days, and could remain anti‐swelling throughout the degradation process, indicating the superior stability of the hydrogels for the long‐term BMI applications. All of the above behaviors are beneficial for the use of PFAPT hydrogels as a brain implant electrode to obtain ECoG signals in clinical applications.

### 2.3 Biocompatible and Anti‐Inflammatory Properties of Hydrogel Electrodes

For clinical BMI application, the biocompatibility and anti‐inflammatory properties are highly required to reduce the accumulation and wrapping around the electrodes, which would lead to the decrease of SNR and recording ability [37]. Firstly, the biocompatibility of the hydrogel electrodes was evaluated by evaluated by seeding PC12 cells on hydrogel electrodes. As shown in Figure S11a, after 1 and 5 d, live/dead assays clearly showed a large number of live cells (green) and few dead cells (red) at hydrogel immersed in TA solution with 2 and 5 mg mL^−1^. The number of dead cells dramatically increased when the concentration TA solution increased to 10 and 15 mg mL^−1^. CCK‐8 was conducted to evaluate the PC12 cells proliferation on hydrogel electrodes (Figure S11b). The number of metabolically active PC12 cells on hydrogel electrodes treated with 2 and 5 mg mL^−1^ TA solution significantly increased with the time of culture and was not statistically different from those on TCPS at the same time points, demonstrating the excellent cytocompatibility. For the overall consideration of adhesive and biocompatible properties, TA solution with 5 mg mL^−1^ was used for post‐treatment of PFAP hydrogels. Then, the inflammation regulation properties were performed using RAW 264.7 macrophages in vitro. As shown in Figure S12, the ratio of CD206/CD68 was statistically increased compared with control group, indicating the promotion of hydrogel electrodes for the polarization of macrophages towards the M2 phenotype. Further, subcutaneous implantation in rats was performed to demonstrate the immunomodulation of our hydrogel electrodes in skin and muscle tissues. As shown in Figure S13, CD3 and CD68 expression after 1‐week implantation were elevated in the hydrogel electrode group compared to control group, indicating a moderate inflammatory response post‐implantation, which is the natural foreign body reaction to implanted materials. After 2‐week implantation, the CD3 and CD68 expression significantly decreased in the hydrogel electrode group with no statical difference compared with control group, indicating the immunomodulation ability of our hydrogel electrodes. The results of fibrosis analysis, such as collagen I and α‐SMA staining, did not display significant differences between the hydrogel electrode and control groups after 2‐week implantation, indicating that our electrode does not induce excessive fibrotic remodeling in either skin or muscle tissues (Figure S14). All the results demonstrate the excellent biological properties of our hydrogel electrodes for BMI applications.

**FIGURE 4:**
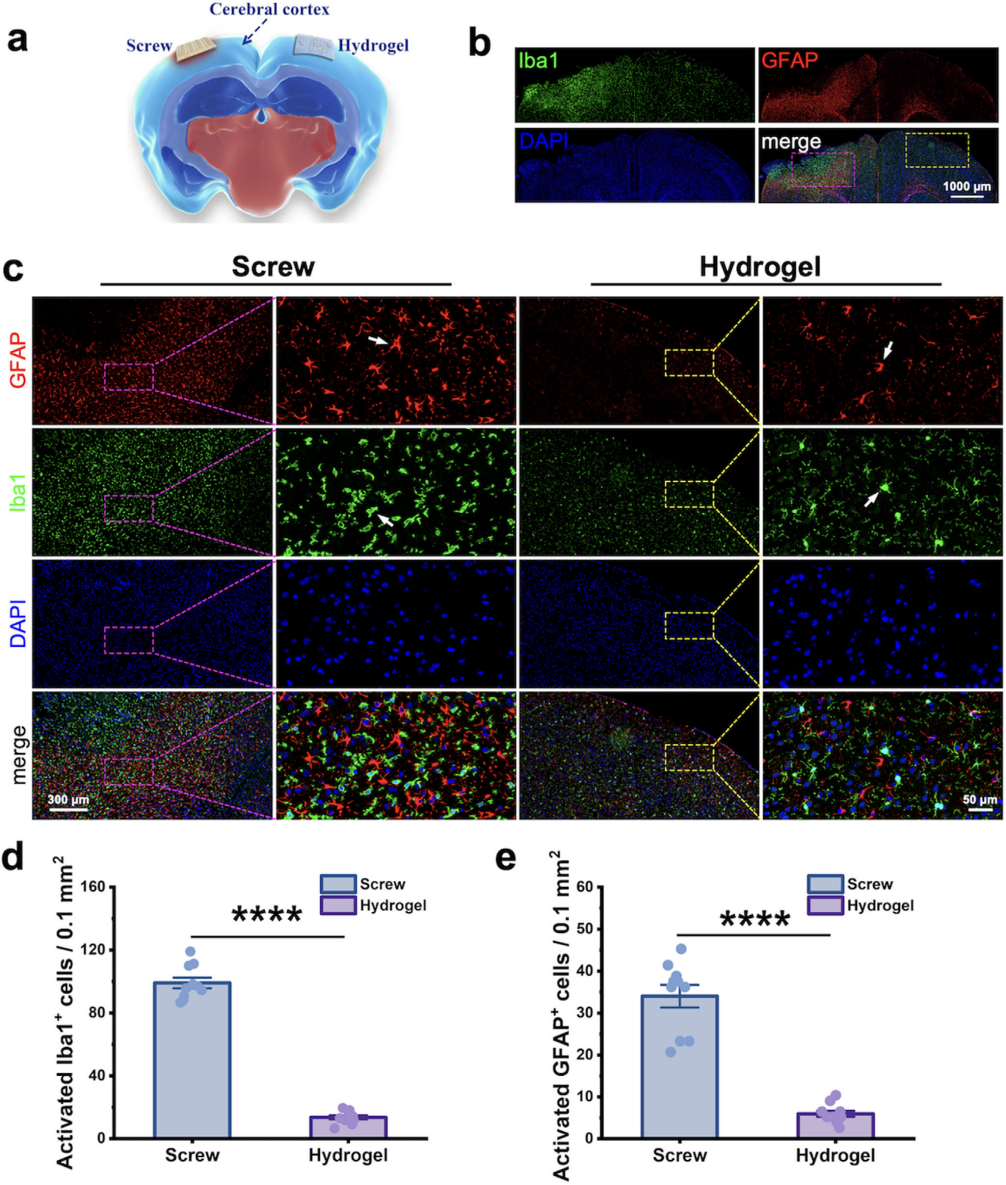
Comparison of glial activation between hydrogel and screw electrodes. (a) Schematic diagram of the electrodes implanted on the brain. (b) Representative confocal images of glial fibrillary acidic protein (GFAP) and Iba1 labeling after 1‐month implantation. (c) Magnified confocal images of GFAP and Iba1 in cerebral cortex areas. (d, e) Quantification of reactive astrogliosis and microgliosis in cerebral cortex areas.

The neuroinflammatory response triggered by implantation of BMI can activate microglia, which further release inflammatory cytokines, thereby exacerbating local inflammatory reactions. The activation of microglia and the release of inflammatory cytokines can affect the ability of the BMI to capture and interpret ECoG signals. To assess the in‐situ biocompatibility and inflammatory response of hydrogel electrodes, the hydrogel electrode and commercially available screw were implanted on the left and right prefrontal cortex in the same rat, respectively (Figure 4a). After 1‐month epidural electrode implantation, the expression of glial fibrillary acidic protein (GFAP), a marker of astrocyte activation, and ionized calcium‐binding adaptor molecule 1 (Iba1), a marker of microglial activation, of hydrogel electrode in both universal (Figure 4b) immunofluorescent staining were much lower than that of the screw group. The magnified images showed that the hydrogel electrode group displayed significantly lower Iba1 expression, with microglia maintaining a more ramified, resting‐state morphology, indicating that the hydrogel attenuated the glial inflammatory response implantation (Figure 4c). Quantitative analysis confirmed a substantial reduction in Iba1l^+^ and GFAP^+^ activated microglia in the hydrogel group relative to the screw group, further confirmed the anti‐Inflammatory of hydrogel electrodes (Figure 4d,e). These results suggested that implantation of the hydrogel reduced neuroinflammation inflammatory response in the cerebral cortex, which is beneficial for the long‐term monitoring ECoG signals.

### 2.4 Acute In Vivo ECoG Signals Recording Performance of the Hydrogel Electrodes

**FIGURE 5:**
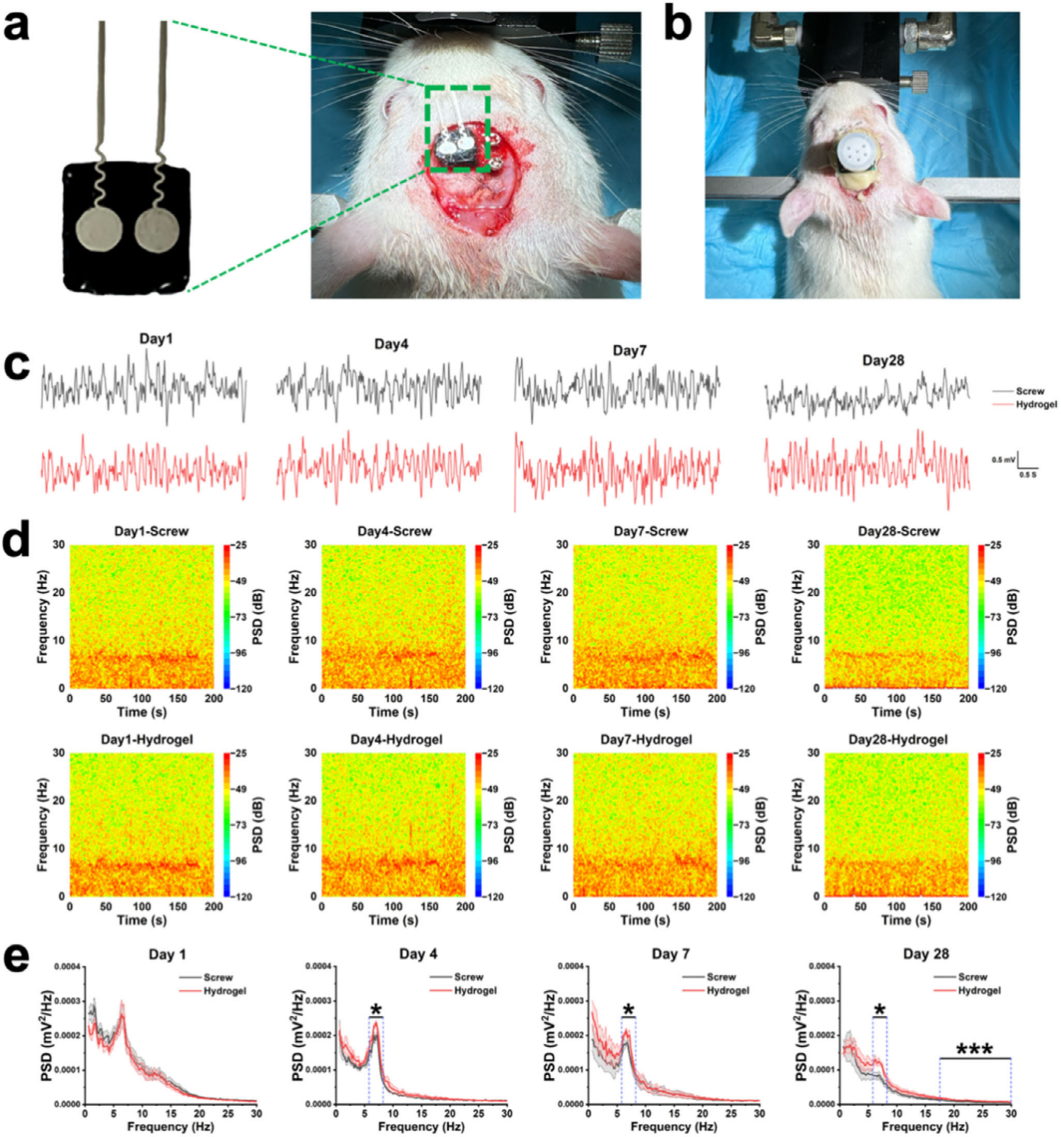
Comparison of the performance between hydrogel electrodes and skull screw electrodes in ECoG acquisition. (a) Photograph of the hydrogel‐integrated BMI. (b) The photograph of surgery of hydrogel electrodes and commercial screw electrodes. (c) ECoG signals collected on days 1, 4, 7, and 28 post‐surgery for hydrogel electrodes and skull screw electrodes. The curves were processed with a 30 Hz low‐pass filter. (d) The time‐frequency spectrograms of ECoG signals collected on days 1, 4, 7, and 28 post‐surgery for hydrogel electrodes and skull screw electrodes. (e) The power spectral density (PSD) of ECoG signals collected on days 1, 4, 7, and 28 post‐surgery for hydrogel electrodes and skull screw electrodes. * indicates *p* < 0.05, and ** indicates *p* < 0.01.

Accurate and stable acquisition of ECoG signals is the primary property for the integrated diagnosis and treatment of BMI. The in vivo ECoG signals recording performance of the hydrogel electrodes was evaluated by comparing the hydrogel electrodes with the commercially available skull screw electrodes at different post‐implantation time points. As shown in Figure 5a and Figure S15, the hydrogel was integrated with a 3D printed Ag electrodes by transferring process from a polydimethylsiloxane substrate to form a mechanical, electrical, and biological coupling BMI, which was then used to record in vivo ECoG signals. The in vivo ECoG signals collected on post‐implantation at day 1, 4, 7, and 28 using hydrogel electrodes and skull screw electrodes, respectively (Figure 5b and Figure S16). The signals were processed with a 30 Hz low‐pass filter to remove high‐frequency noise. From the waveforms, it can be observed that the signals collected by the hydrogel electrodes maintained a high signal‐to‐noise ratio (SNR) and stability even at day 28, whereas the signal quality of the skull screw electrodes gradually declined, indicating the advantages of hydrogel electrodes in long‐term use (Figure 5c). Moreover, time‐frequency spectrum analysis was performed to illustrate the spectral distribution of ECoG signals collected by the two electrodes at different time points. As shown in Figure 5d, the hydrogel electrodes maintained clear spectral characteristics throughout the post‐implantation period, while the spectral features of the skull screw electrodes became increasingly blurred in the later stages, particularly at day 28. The energy intensity of δ, θ, α, and β bands in ECoG signals collected by hydrogel electrodes is comparable to the skull screw electrodes during the whole implantation, which indicates that hydrogel electrodes are more effective in capturing prominent features of ECoG during long‐term use (Figure S17). To further validates the superior performance of hydrogel electrodes in long‐term signal acquisition, especially in θ bands, power spectral density (PSD) analysis is widely used to reveal the energy distribution and interrelationship of signals. As shown in Figure 5e and Figure S18, the PSD curves in different frequency bands of the hydrogel electrodes were statically higher than that of the skull screw electrodes in *θ* bands from 4 to 28 d, indicating better stability and reliability in long‐term signal acquisition. All the results suggested the ability of the hydrogel electrodes for accurately recording ECoG signals, which is fundamental for closed‐loop BMI applications.

### 2.5 The Application of Hydrogel Electrode for Diagnosis of SNI Model

**FIGURE 6:**
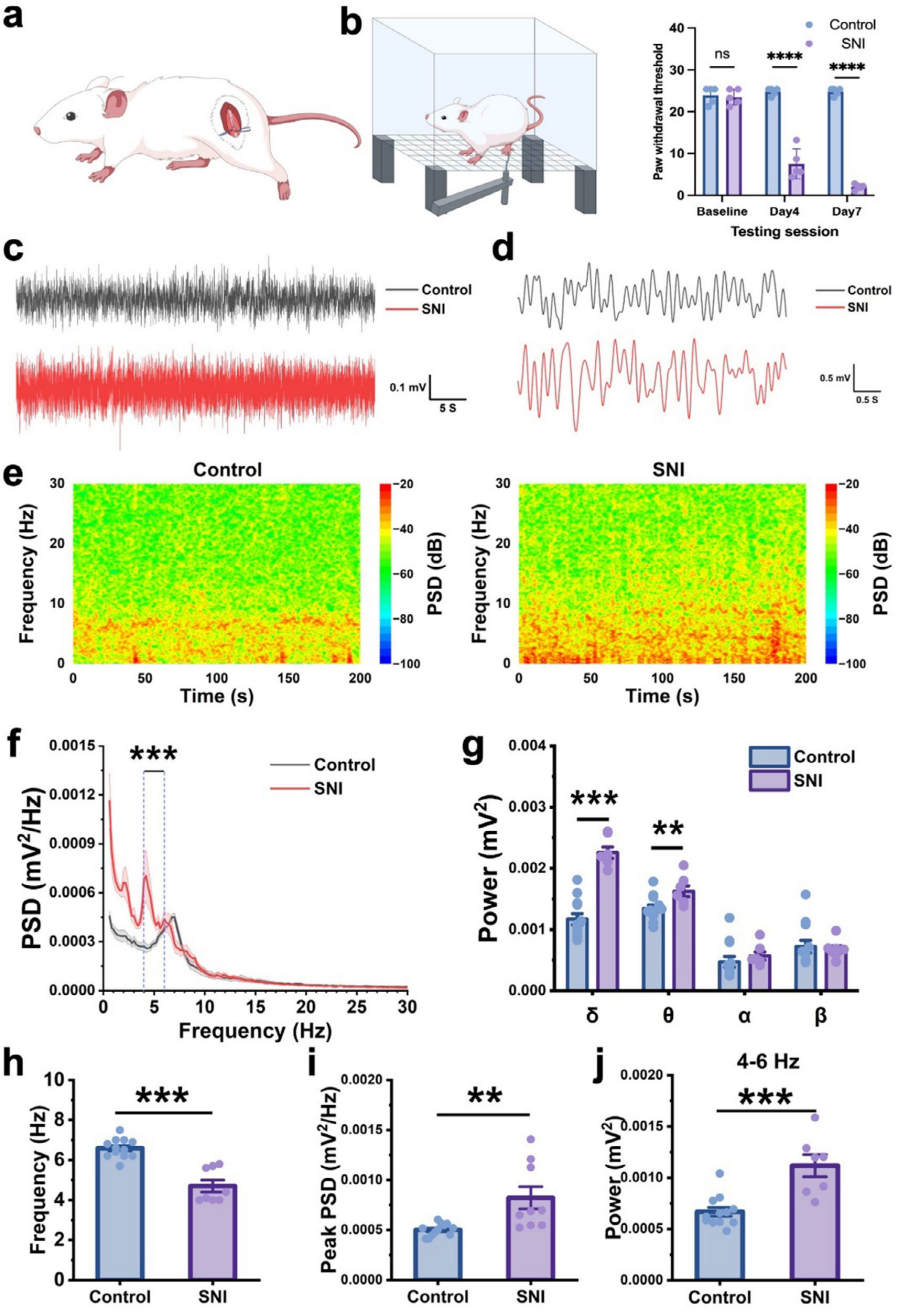
ECoG analysis of SNI and control rats recorded at day 7 after hydrogel electrode implantation. (a) Schematic diagram of spared nerve injury rat. (b) Schematic diagram and quantitative result of paw withdrawal threshold test. (c) Representative ECoG signals of SNI and control rats. (d) ECoG curves of SNI and control mice after an 8 Hz low‐pass filter. (e) Time‐frequency spectrograms of EEG signals from SNI and control rats. (f) PSD of ECoG signals from SNI and control rats, with the blue dashed lines marking frequencies from 4 to 6 Hz. (g) Energy intensity of δ, θ, α, and β bands in the ECoG signals of SNI and control rats. (h) Frequencies corresponding to the peaks in (f). Peak values (i) and energy intensity (j) corresponding to the 4 – 6 Hz range within the blue dashed lines in (f).

To validate the acquisition property of ECoG signals of hydrogel electrode for NP, a spared nerve injury (SNI) animal model was established (Figure 6a). Figure 6b compares the paw withdrawal thresholds of SNI and control rats. At days 4 and 7, the paw withdrawal thresholds to mechanical stimuli of SNI rat were significantly reduced, indicating the successful establishment of the SNI model. Figure 6c presents the analysis of ECoG signals recorded 7 d after hydrogel electrode implantation in rat with the SNI model and control group. Compared to the control group, the ECoG signals of SNI group exhibited higher amplitudes and more pronounced fluctuations, suggesting that NP may lead to abnormally enhanced neural activity. After applying an 8 Hz low‐pass filter to the ECoG signals, the low‐frequency signal amplitudes of SNI rat were significantly higher than those of the control group (Figure 6d). In the time‐frequency spectrogram, the SNI group showed higher energy density in the low‐frequency range compared to control group (particularly in the δ and θ bands), indicating that NP may significantly enhance low‐frequency neural activity (Figure 6e). The same tendency was also found in PSD data. In Figure 6f, PSD values in the 4–6 Hz range were significantly higher in SNI rat compared to that of control group. The result of energy intensity across frequency bands shows the δ and θ band energy intensities were significantly higher in SNI rat compared to the control group, while no significant differences were observed in the α and β bands, consistent with the results of clinical result (Figure 6g). It worth to note that δ band is mainly associated with deep sleep and suppression of brain function, which lack of specificity required for pain management. We further analyze the peak frequency of the ECoG in θ bands. As shown in Figure 6h, frequencies corresponding to the peaks exhibited statically difference range from 4 to 6 Hz in the control and SNI groups. The power and peak PSD of SNI group in the range of 4 to 6 Hz also exhibited significant difference compared to control group (Figure 6i,j). These results demonstrate the ability of our hydrogel electrodes for diagnosis of NP. Furthermore, the variation of θ bands in NP animal model was consistent with clinical results, which reconfirmed the θ bands can serve as an objective biomarker for NP.

### 2.6 Pain Management Based on Electrical Stimulation of Hydrogel Electrodes

**FIGURE 7:**
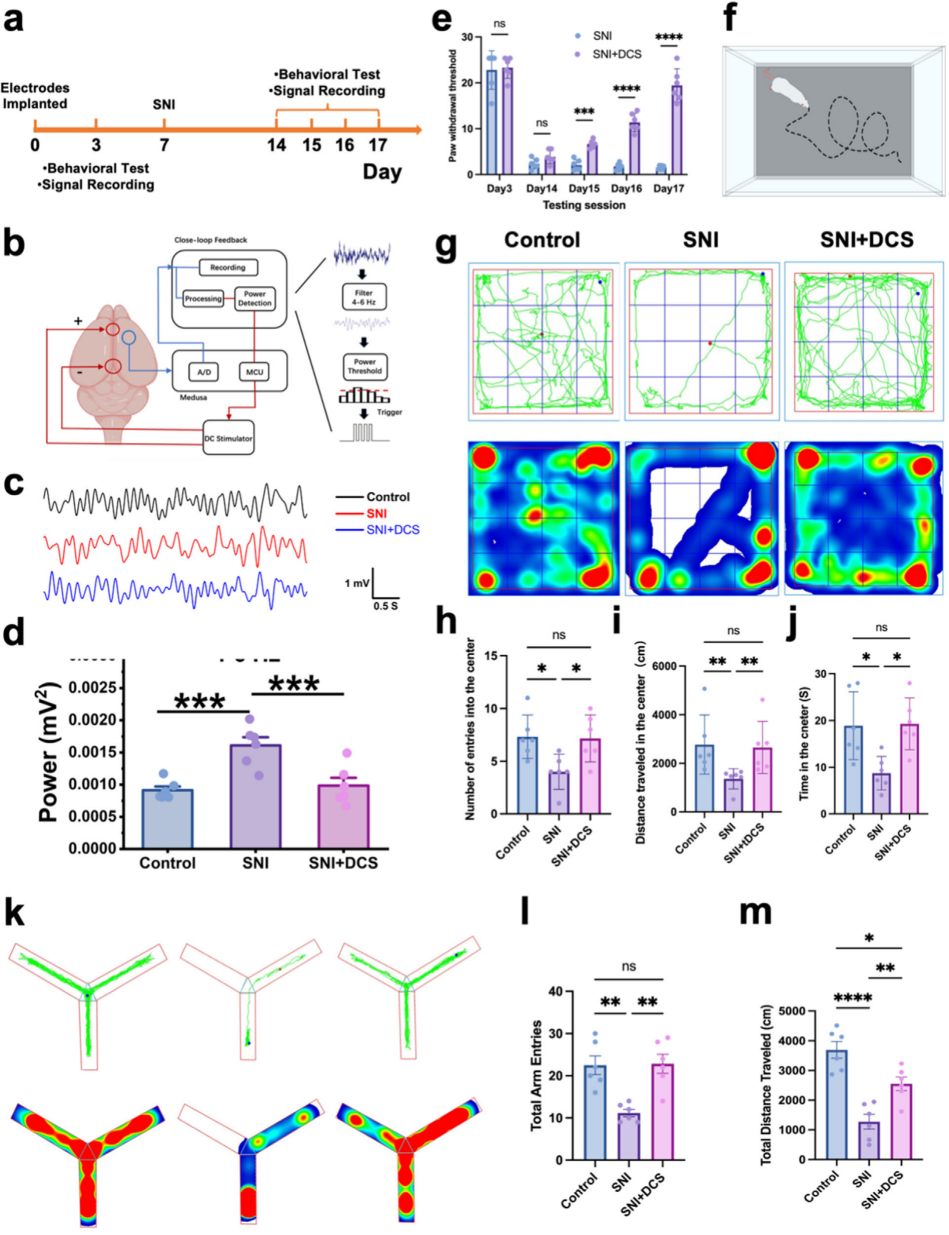
Improvement in pain and depression‐like behaviors in rats after electrical stimulation. (a) Schematic diagram of the experiment design. (b) Schematic diagram of the closed‐loop system: The system continuously records neural signals, processes them to detect pain‐associated oscillatory patterns (e.g., θ band), and provides precise electrical stimulation based on pre‐defined thresholds. (c) Representative ECoG waveforms of control, SNI, and SNI+DCS. (d) Comparison of energy intensity in 4–6 Hz of ECoG signals of control, SNI, and SNI+DCS. (e) Comparison of pain withdraw threshold of SNI and SNI+DCS groups. (f, g) The schematic diagram and trajectory plot of spontaneous activity in open field test. (h–j) Quantitative analysis of open field tests. (k) Trajectory plot of spontaneous activity in Y maze test. (l, m) Quantitative analysis of Y maze test.

The therapeutic effects of electrical stimulation were systematically evaluated through behavioral tests and neural signal analysis [38]. Figure 7a illustrates the effects of electrical stimulation on alleviating pain and depression‐like behaviors in SNI model rats. At day 0, hydrogel electrodes were implanted, and baseline behavioral tests and signal recordings were conducted at day 3. At day 7, SNI surgery was performed. From days 14 to 17, behavioral tests and signal recordings were conducted to evaluate the therapeutic effects of electrical stimulation. The experiment included three groups: control, SNI (without electrical stimulation), and SNI+DCS (with direct current stimulation). Figure 7b illustrate the approach of closed‐loop electrical stimulation system including signal recording and stimulating process. Figure 7c,d shows representative ECoG waveforms of different groups. ECoG signals in the control group exhibited stable fluctuations with low amplitudes, while ECoG signals showed significantly increased amplitudes and more pronounced fluctuations in SNI group, indicating abnormally enhanced neural activity due to NP. In the SNI+DCS group, ECoG signals became more stable. And the amplitudes were significantly reduced after electrical stimulation, demonstrating effective modulation of abnormal neural activity. Paw withdrawal threshold test was performed to further assess the pain management of electrical stimulation (Figure 7e). From postoperative days 14 to 17, the paw withdrawal thresholds of SNI group significantly elevated with increased time of electrical stimulation. At day 17, the paw withdrawal thresholds of SNI+DCS group approached to the preoperative value. The open field test was used to assess exploratory behavior and anxiety levels in rats (Figure 7f). Rats were allowed to move freely in an open square arena, and their movement trajectories and behavioral characteristics were recorded. Figure 7g–j shows the movement trajectories (top) and heatmaps (bottom) of these three groups in the open field test. Control group exhibited evenly distributed movement trajectories, with heatmaps showing frequent activity in the central area. The movement trajectories of SNI group were primarily concentrated in the peripheral area, with heatmaps indicating reduced activity in the central area, suggesting elevated anxiety levels. SNI+DCS group exhibited movement trajectories and heatmap distributions similar to the control group, indicating that electrical stimulation significantly alleviated anxiety‐like behaviors. The Y maze test was used to assess exploratory behavior and depression‐like traits (Figure 7k). As shown in Figure 7l,m, control group exhibited evenly distributed movement trajectories, with heatmaps showing balanced activity between the open and closed arms. The movement trajectories of SNI group were primarily concentrated in the closed arms, with heatmaps showing significantly reduced activity in the open arms, suggesting increased depression‐like behaviors. SNI+DCS group exhibited movement trajectories and heatmap distributions similar to the control group, indicating that electrical stimulation significantly alleviated depression‐like behaviors.

**FIGURE 8:**
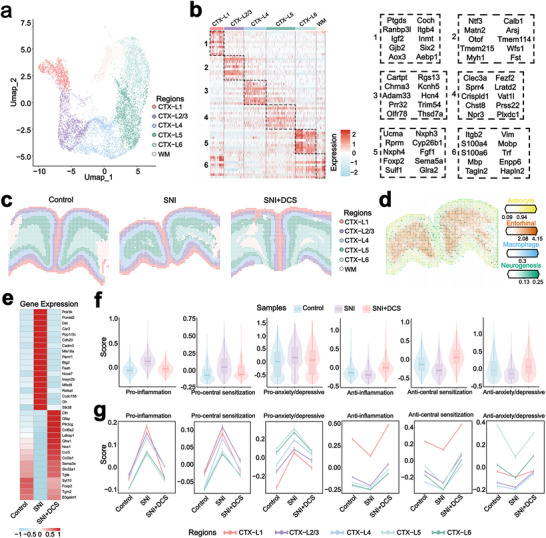
(a) UMAP visualization of dimensionality‐reduced spatial transcriptomic data, with unsupervised clustering identifying six distinct regions. (b) Annotation of six prefrontal cortical (CTX) regions using classical layer‐specific marker genes. (c) Spatial distribution mapping of the annotated regions onto the tissue architecture. (d) Deconvolution analysis (Cell2location) integrating single‐cell reference data, visualizing spatial abundance patterns of four major cell types. (e) Differentially expressed genes (DEGs) across three experimental groups (Sham, SNI, SNI+DCS), highlighting genes significantly upregulated or downregulated in the SNI group. (f) Global pathway activity scoring for the three groups using predefined gene sets. (g) Region‐specific pathway activity scoring across individual cortical layers.

To further investigate the mechanism of the DCS‐mediated modulation of chronic pain‐related pathways, the molecular heterogeneity and cellular dynamics across cortical layers under different experimental conditions were systematically dissected using spatial transcriptomics. First, unsupervised clustering analysis (UMAP) partitioned the cortical tissue into six molecular layers (CTX‐L1 to CTX‐L6), with layer‐specific molecular identities defined by classical marker genes (e.g. Foxp2 marking CTX‐L5) (Figure 8a,b). Spatial mapping confirmed anatomical coherence, by which CTX‐L1 localized to superficial layers while CTX‐L6 resided in deeper regions, with gene expression profiles aligning precisely with laminar positions (Figure 8c). Furthermore, deconvolution analysis using Cell2location, integrated with single‐cell reference data, revealed the spatial distributions of key cell types. Specifically, astrocytes were significantly enriched in CTX‐L1, while entorhinal cells exhibited specific accumulation in CTX‐L5 (Figure 8d).

Differential gene expression analysis and pathway activity scoring revealed dynamic changes across treatment groups (Figure 8e–g). Compared to the control group, the SNI group exhibited significant upregulation of pro‐inflammatory genes (e.g., Car3, Cdh20), pro‐central sensitization genes (e.g., Insyn2b, Stk38), and anxiety‐promoting genes (e.g., Ncoa7), alongside downregulation of anti‐inflammatory genes (e.g., Cfh, Gfra1), anti‐anxiety/depressive genes (e.g., Nos1), and anti‐central sensitization genes (e.g., Slc32a1). Notably, SNI+DCS intervention suppressed expression of pro‐inflammatory, pro‐sensitization, and anxiety‐promoting genes while reversing the downregulation trends of anti‐inflammatory and anti‐anxiety genes. These results indicate that DCS partially reverses SNI‐induced molecular pathology by bidirectionally regulating inflammation, central sensitization, and affective pathways. Region‐specific scoring (Figure 8g) further delineated laminar effects: CTX‐L1 showed heightened neuroinflammation in SNI, while CTX‐L5 demonstrated significant restoration of affective comorbidity pathway regulation (e.g., serotonin signaling) post‐DCS. In summary, this work not only identifies layer‐specific molecular responses in chronic pain (e.g., CTX‐L1 as an inflammation hotspot; CTX‐L5 regulating affective phenotypes) but also elucidates how DCS alleviates central sensitization and emotional comorbidities by targeting discrete cortical layers (e.g., suppressing CTX‐L1 inflammation; enhancing CTX‐L5 anti‐anxiety pathways). This spatially resolved molecular atlas provides a critical framework for precision intervention in pain‐related neural circuits.

## 3 Conclusion

This study elucidates the potential of integrating low‐frequency oscillatory biomarkers and hydrogel‐based neural interfaces for diagnosing and managing NP. The clinical finding of low‐frequency oscillations, particularly the *θ* band, as specific and robust neuro‐biomarkers of NP for the first time, offering critical insights into NP's neurophysiological mechanisms and establish a framework for future clinical translation. The developed mechanical‐electrical‐biological coupled PFAPT hydrogel electrode marks a significant advancement in for NP research, as chronic conditions require long‐term monitoring to understand pain dynamics and treatment responses. The integration of hydrogel electrodes into a closed‐loop BMI system demonstrated bidirectionally regulating inflammation, central sensitization, and affective pathways, achieving precise and demand‐based pain relief. Moreover, this study provides a proof‐of‐concept in preclinical models, translating these findings to human clinical settings poses challenges, providing a new research paradigm for the integrated diagnosis and therapeutics of related diseases.

## Author Contributions

**Yun Ji**: software, validation, supervision, investigation, data curation, formal analysis, writing – original draft, writing – review and editing, methodology, project administration. **Tao Li**: conceptualization, investigation, supervision, formal analysis, visualization. **Xiuqian Guo**: investigation. **Shutao Zhao**: methodology, visualization. **Ke Ma**: resources, validation, funding acquisition, supervision, project administration. **Guoqiang Lei**: investigation, software, methodology, resources, writing – review and editing. **Huichun Luo**: conceptualization, data curation, supervision, funding acquisition. **Tao Shi**: investigation. **Jiao Xiang**: investigation. **Jiayun Wu**: methodology, visualization. **Weitang Liu**: conceptualization, supervision. **Yuxin Zhang**: methodology, validation. **Wangao Zhang**: methodology, data curation. **Wenhui Liu**: visualization. **Chuanglong He**: funding acquisition, resources. **Tao Wu**: resources, funding acquisition. **Shuo Chen**: resources, funding acquisition. **Xiaoyu Liao**: validation, methodology.

## Ethics Statement

This study involved a human clinical trial component. The trial was prospectively registered at ClinicalTrials.gov (registration number: NCT06290024). The study was approved by the Ethics Committee of Xinhua Hospital, Shanghai Jiaotong University School of Medicine (approval number: XH‐23‐013). All participants provided written informed consent prior to enrollment. All procedures were performed in accordance with the Declaration of Helsinki.

## Conflicts of Interest

The authors declare no conflicts of interest.

## Supporting information




**Supporting File**: advs76269‐sup‐0001‐SuppMat.docx.

## Data Availability

The data supporting the findings of this study can be made available by the corresponding author upon reasonable request.
