# Supplementary material for: Closed‐Loop Decoding and Intervention of Pain: A Novel BMI Strategy Integrating θ‐Band Detection and Mechano‐Electro‐Biological Coupled Hydrogels
Source: Adv Sci (Weinh). 2026 Jun 26:e76269. Online ahead of print. doi: 10.1002/advs.76269 (PMC13336839; doi:10.1002/advs.76269)
Supplement: Supplementary file 1 — Supporting File: advs76269‐sup‐0001‐SuppMat.docx. [file ADVS-9999-e76269-s001.docx]

**Closed-Loop Decoding and Intervention of Pain: A Novel BMI Strategy Integrating θ-Band Detection and Mechano-Electro-Biological Coupled Hydrogels**

Yun Ji^1^^†^, Tao Li^2†^, Guoqiang Lei^3†^, Huichun Luo^4†^, Xiuqian Guo^1^, Jiao Xiang^5^, Tao Shi^6^, Weitang Liu^7^, Yuxin Zhang^8^, Xiaoyu Liao^9^, Shutao Zhao^10^, Jiayun Wu^11^, Wangao Zhang^12^, Wenhui Liu^13^ Chuanglong He^3^*, Shuo Chen^3^*, Tao Wu^14, 15^*, Ke Ma^1,16^*

^1^ Department of Pain Medicine, Xinhua Hospital Affiliated to Shanghai Jiao Tong University, Shanghai 200092, China; ^2^ Department of orthopedics School of Medicine, Xin Hua Hospital Affiliated to Shanghai Jiao Tong University, Shanghai 200092, China; ^3^ State Key Laboratory of Advanced Fiber Materials, College of Biological Science and Medical Engineering, Donghua University, Shanghai 201620, China; ^4^ Department of Anesthesiology, Renji Hospital Affiliated to Shanghai Jiao Tong University, Key Laboratory of Anesthesiology (Shanghai Jiao Tong University), Ministry of Education, Shanghai 200127, China; ^5^ Department of Pain, The Second Affiliated Hospital of Anhui Medical University, Hefei 230601, China; ^6^ Department of Pain, The Second Affiliated Hospital of Kunming Medical University, Kunming 650101, China; ^7^ Institute of Infectious Disease and Biosecurity, Fudan University, Shanghai 200032, China; ^8^ Department of Oral Surgery, Shanghai Ninth People’s Hospital Affiliated to Shanghai Jiao Tong University, Shanghai 200011, China; ^9^ Department of Rehabilitation Medicine, The Affiliated Suzhou Hospital of Nanjing Medical University, Suzhou Municipal Hospital, Suzhou 215008, China; ^10^ Institute for Developmental and Regenerative Cardiovascular Medicine, MOE-Shanghai Key Laboratory of Children's Environmental Health, Xin Hua Hospital Affiliated to Shanghai Jiao Tong University, Shanghai 200092, China; ^11^ Shanghai Jiao Tong University School of Medicine, Shanghai 200025, China; ^12^ Department of Pain Medicine, First Affiliated Hospital of Anhui University of Chinese Medicine, Hefei, 230038, China; ^13^People’s Hospital Affiliated to Fujian University of Traditional Chinese Medicine, Fuzhou 350004, China; ^14^ School of Health Science and Engineering, University of Shanghai for Science and Technology, Shanghai 200093, China; ^15^ Centre for Collaborative Research, Shanghai University of Medicine and Health Sciences, Shanghai 201318, China.^16^ Department of Pain Medicine, Ninth People's Hospital Affiliated to Shanghai Jiao Tong University School of Medicine ,Shanghai 200011, China

†These authors contributed equally to this work.

*Corresponding author.

Email: hcl@dhu.edu.cn; schen@dhu.edu.cn; wutao0324@shsmu.edu.cn; marke72@sjtu.edu.cn

**Supplemental Information**

1. **Preparation and characterization of PFAPT hydrogel.**

**1.1 Materials**

Pluronic F127 and sodium alginate(SA) were purchased from Macklin China Corporation. PEDOT: PSS aqueous solution (Clevios PH1000) with a solid content around 1.15 wt% were obtained from Heraeus Electronic Materials. Tannic acid(TA) was provided by Sigma. Lithium Phenyl(2, 4, 6-trimethylbenzoyl) phosphinate (LAP) were bought from Macklin China Corporation. All reagents were used without further treatment.

**1.2 Synthesis of Methacrylated Alginate (AlgMA)**

Briefly, SA was dissolved in water to prepare a 2% (w/v) SA aqueous solution, and then methacrylic anhydride was added to the SA aqueous solution, and the molar ratio of anhydride/-OH was set to 10:1. During anhydride esterification, a 5 M NaOH solution is gradually added to maintain the pH of the polymer solution at 8. After 24 h of incubation at 0°C, the mixture is precipitated in ethanol and washed several times with ethanol to remove excess methacrylic anhydride and methacrylic acid. Finally, AlgMA was obtained after vacuum drying at 40^o^C. The chemical structure of AlgMA was confirmed by ^1^H NMR (Supplementary Fig. S3) spectra using D_2_O as solvent.

**1.3 Synthesis of Pluronic F127- diacrylate (PF127-DA)**

PF127-DA is prepared by acrylicylation of PF127 with acryloyl chloride. A volume of 2.54 g (0.2 mmol) of PF127 was dissolved in 20 mL anhydrous dichloromethane in an ice bath and degassed by pouring nitrogen for 20 minutes. Then, 0.084 mL of triethylamine (0.6 mmol) and 0.05 mL of acryloyl chloride (0.6 mmol) were slowly injected into the above solutions in a nitrogen environment. After the reaction was performed at room temperature for 24 hours, the solvent was removed by rotary evaporation, the crude product was dissolved in distilled water, and thoroughly dialyzed with deionized water (MWCO 3500) for three days. Pure products are obtained by lyophilization. The chemical structure of PF127-DA was confirmed by ^1^H NMR (Supplementary Fig. S2) spectra using CDCl_3_ as a solvent.

**1.4** **Preparation of mechanical-electrical-biological coupling hydrogel (PFAPT):**

First, PF127-DA, AlgMA and LAP were dissolved in deionized water, and then simply mixed with PEDOT:PSS aqueous solution to obtain a precursor solution. Ice baths are required for the preparation of precursor solutions to facilitate the solubilization of PF127-DA. The precursor solution should be stored at 4^o^C in the dark. The solution is then injected into a special mold and solidified in situ into PFAP Hydrogel by UV irradiation (365 nm, 100 mW cm^–2^) for 30 seconds. Subsequently, the cured hydrogels were immersed in a large volume of deionized water and placed on an orbital shaker at room temperature. To ensure the complete diffusion and removal of unreacted residues and excess LAP, the deionized water was refreshed every 6 hours over a total washing period of 24 hours. Then, the hydrogels were soaked in TA solutions for 2 hours to obtain the PFAPT hydrogel. The resulting PFAPT Hydrogel is abbreviated as PFA_x_P_y_T hydrogel (PF: PF127-DA, A: AlgMA, P: PEDOT:PSS), where “x” represents the different dosages of ALGMA and “y” denotes the different dosages of PEDOT:PSS. For details, see Table S1.

**1.5 Transmission electron microscope and scanning electron microscopy:**

The morphologies of the PF127-DA micelles were observed using a field emission transmission electronic microscope (FETEM, JEM-2100F, Japan). Briefly, 5 μl of PF127-DA aqueous solution (1 wt.%) was applied to the copper mesh, and the copper mesh was placed under an infrared baking lamp to volatilize the solvent. Samples are observed after drying. The microscopic morphology of PFS_x_P_y_ gel was observed by scanning electron microscopy (SEM, Thermo Scientific Phenom Pharos, USA). The hydrogels are freezed with liquid nitrogen and immediately lyophilized, followed by further sputtering gold plating to improve conductivity for easy observation.

**1.6 Swelling Ratio**

The anti-swelling properties of PFAPT hydrogels were studied by swelling experiments. First, 1 ml of precursor solution is injected into the mold and subjected to ultraviolet irradiation at 37^o^C and 4^o^C to form a cylindrical hydrogel (diameter: 1 cm). The initial hydrogel weight is Wo, and the hydrogel is then soaked in PBS buffer at a constant temperature of 37^o^C and 4^o^C, respectively. After 12, 24, 36, 48, and 60 days of immersion, the swelling hydrogel was taken out, and remove surface moisture with filter paper. The weight of the swollen hydrogel is denoted as Ws, and the swelling rate is calculated using equation (1):

$$Swelling ratio=\frac{(Ws-Wo)}{Wo}\times100\% (1)$$

**1.7 Mechanical performance and adhesion performance tests:**

To measure the mechanical properties of PFAPT hydrogels, a uniaxial tensile experiment was conducted using an MTS E42 universal testing machine at 25^o^C. The hydrogels were cast into dumbbell-shaped samples using polytetrafluoroethylene molds. During the experiment, the tensile strength and fracture strain were tested with a constant extension speed of 50 mm min^–1^.

To measure the adhesion strength, PFAPT hydrogel samples with an adhesion area of 2 × 2 cm were prepared and subjected to a standard lap shear strength test using an MTS E42 universal testing machine at a constant tensile speed of 50 mm min^-1^. The lap shear strength was calculated by dividing the maximum force by the adhesion area.

**1.8 Measurement of the electrical performance:**.

To measure the electrical conductivity of PFAPT hydrogels, resistance were recorded by a Keithley 2700 digital multimeter. The electrical conductivity (σ) of all the hydrogel samples was calculated according to the following equation:

$$\sigma=\frac{L}{WRT} (2)$$

where L, W, R, and T are the length, width, resistance, and thickness of the samples.

The electrochemical tests such as electrochemical impedance (EIS) and cyclic voltammetry (CV) are performed in potentiostats (CH Instruments 610F) with standard three-electrode systems. All the working electrodes are PFAPT hydrogels (immersion size: 2 × 5 × 1 mm) clamped with Pt electrode clamps, meanwhile, a bare Pt electrode (size: 10 × 15 × 0.1 mm) and an Ag/AgCl wire were used as the counter electrode and reference electrode, respectively. Standard PBS solution (0.01 mol L^-1^, pH =7.1 - 7.4) was used as the electrolyte solution. For EIS measurements, the PFAPT coatings were tested at the frequency range from 10^0^ Hz to 10^5^ Hz in order to evaluate the interfacial impedance.

The CV measurements were accomplished by cycling 20 times at a constant scan rate of 100 mV s^-1^ over a potential range of -0.5 ~ +0.5 V. The charge storage capacitance (CSC) values for different samples were calculated by the following equation:

$$CSC=\int_{E_{i}}^{E_{t}} \frac{i(E)}{2vA}dE \left( 3 \right)$$

where i represents the current at each potential, E_i_ and E_t_ represent the starting and final potentials, v represents the potential scanning rate, and A is the area of the PFAPT hydrogel immersed in standard PBS buffer.

**1.9 Preparation of flexible electrode based on PDMS film**

A flexible electrode was produced using a microelectronic printer (MP1200, Shanghai Mifang Electronic Technology Co., Ltd, Shanghai, China). Once the conductive ink was removed from the refrigerator, it was allowed to sit at ambient temperature (approximately 25^o^C) for more than an hour to ensure proper mixing. Afterward, it was gently stirred for 15 minutes. This microelectronic printer operates by extruding functional inks through a small nozzle under pneumatic control. The ink droplets are continuously deposited onto a flexible substrate, forming a conductive path once dried for a certain duration. Factors such as air pressure, printing speed, the gap between the nozzle and substrate, and needle diameter all influence the printing outcomes, including line width, thickness, and uniformity. Finally, the electrodes were baked in a Precision Air Blast Drying Oven (BPG-9106A, Shanghai Yiheng Scientific Instrument Co., Ltd., Shanghai, China) at 100^o^C for 30 minutes to attain optimal electrical performance.

The silver/silver chloride conductive ink was printed onto a PDMS film to create printed electrodes, which were then combined with PEDOT:PPS hydrogel to form flexible EEG bioelectrodes. The hydrogel adheres to the skin, while the conductive side of the silver/silver chloride electrode is in contact with the hydrogel. Thus, the collected EEG signals are transmitted to peripheral devices via the composite electrodes. Meanwhile, the PDMS film provides robust mechanical support for the electrodes, ensuring durability and flexibility.

**2. Methods**

**2.1. Clinical study**

Both neuropathic pain (NP) patients and health control (HC) participants were recruited. Inclusion criteria for NP patients were as follows: (1) age from 40 to 80; (2) pain lasting at least one month; (3) No other major neurological disease or psychiatric disorders. As shown in Table S2, demographic and clinical characteristics were compared between the NP group and the HC group. Pain intensity was measured using the Visual Analog Scale (VAS), a 0–10 scale where 0 indicates no pain and 10 indicates the worst possible pain. The Douleur Neuropathique 4 questions (DN4) is a validated screening tool for neuropathic pain, with a total score ranging from 0 to 10; a score of ≥4 suggests the presence of neuropathic pain. Sleep quality over the past month was assessed by the Pittsburgh Sleep Quality Index (PSQI), which has a global score range of 0–21, with higher scores indicating poorer sleep quality (a score >5 is commonly defined as poor sleep). Depressive symptoms were evaluated using the Patient Health Questionnaire-9 (PHQ9),a 9-item scale ranging from 0 to 27; higher scores reflect more severe depression. Anxiety symptoms were measured with the Generalized Anxiety Disorder 7-item scale (GAD7), where total scores range from 0 to 21 and higher scores indicate greater anxiety severity. Education years refer to the total number of years of formal education received. Age is presented as mean ± standard deviation (SD). Statistical comparisons between groups were performed using independent t‑tests for continuous variables and the chi‑square (χ²) test for sex distribution, with p < 0.05 considered statistically significant.

The study was approved by the Ethics Committee of Xinhua Hospital, Shanghai Jiaotong University School of Medicine (approval number: XH-23-013),The clinical research registration number is NCT06290024. All participants signed an informed consent before the experiment.

The clinical information of the NP patients was collected by questionaries and the pain intensity was assessed by visual analog scale (VAS). A five-minute resting high-density EEG was recorded with eyes closed (128-channel EEG Geodesic Net Amps, Electrical Geodesics, Inc., Eugene, OR). The sampling rate of EEG data was 500 Hz and a high-pass filter of 0.01 Hz was applied when recording. The impedances of the scalp electrodes were lower than 50 KΩ. When recording, the participants were seated in a suitable chair and asked to remain calm but not fall asleep.

**2.2 EEG data preprocessing and analysis**

EEG preprocessing is aimed at removing noise, improving signal-to-noise ratio, and providing a reliable data foundation for subsequent analysis. It includes: (1) a 1 Hz high-pass filter to remove linear trends; (2) a 50 Hz notch filter to remove line noise; (3) a 45 Hz low-pass filter; (4) topographically interpolated bad channels using spherical splines, and re-referenced to the grand average; (5) artifactual components, including ocular, muscle, and cardiac signals, were identified and removed using independent component analysis (ICA); (6) segmenting signal into non-overlapping 2-s epochs excluding that epochs exceed 100 µV in amplitudes. After preprocessing, signals with > 15% bad channels or > 50% artifactual segments were defined as poor-quality data and would not be further analyzed.

The brain neural activity levels were estimated by a fast Fourier transform and the power of each frequency bin estimated as the total power in the frequency range. In this study, we also separately calculated the neural activity of the frontal lobe. The activity of the frontal lobe was represented by the average activities of electrodes 9, 11, 22, 24, and 124.

Between-group comparisons at different frequency bins were performed using cluster-based permutation tests. Monte Carlo p values were calculated on 1000 permutations and clusters were defined as more than two neighboring electrodes with p < 0.05, controlling for multiple comparisons across space for p < 0.025 (two-tailed test). The only frontal areas activities between groups, the independent-samples T-tests were performed. Moreover, the relationship between activities of the frontal lobe and pain intensity of NP patients was evaluated by the Spearman correlation since the distribution of pain intensity did not obey normal distribution. All the preprocessing was performed based on the EEGLAB toolbox and other analyses were performed in MATLAB R2016b (MathWorks, Natick, Massachusetts, USA) with manual scripts.

**3. Animal experiment**

Experiments were conducted on adult male Sprague-Dawley rats (200 ± 20 g), supplied by the Animal Center of Xinhua Hospital, Shanghai. All procedures adhered to the guidelines set by the International Association for the Study of Pain. The animals were housed in a temperature-controlled environment (23^o^C) with a 12-hour light-dark cycle for a 7-day acclimatization period prior to surgery at the Xinhua Hospital animal facility. The approval number for animal ethics is XHEC-F-2024-032

**3.1 Surgical Procedure**

**3.1.1 Spared Nerve Injury (SNI) Model**

Rats were randomly divided into three groups: normal control group (control group), SNI group, and SNI+DCS group. NP was induced using the SNI model. Briefly, rats were anesthetized with isoflurane, and the left common peroneal and tibial nerves were tightly ligated with 5-0 silk sutures, transected distal to the ligation, and 2 - 4 mm of the nerve stump was removed, while leaving the sural nerve intact (n = 6 per group).

**3.1.2 Surgical electrode implantation**

Animals were anaesthetized with gaseous isoflurane throughout the course of surgery and craniotomies were performed with a stereotaxic apparatus (RWD, Shenzhen, China). The skin was shaved and a scalp incision was made over the sagittal midline of the skull and the tissue was retracted to expose the top of the skull. A craniotomy was given to open a 3 × 3 mm bone window for hydrogel electrode placement on in the left primary sensory area (S1, AP: +0.16 mm, ML: +3.60 mm; the atlas of Paxions and Watson). One stainless-steel screw was implanted in the skull contralaterally as conventional recording electrode for signal comparation. Six screws were drilled through the skull prior to craniotomy, to stabilize and strengthen the implant, two of which above the cerebellum served as ground and reference electrodes. The craniotomy was then sealed with sterile bone wax. The exposed skull area was then built up with dental cement all the way from the skull surface up to the base of the electrode connector to provide stability and long-term retention of the implant.

For close-loop experiment, hydrogel electrode was placed on the same location (S1, AP: +0.16 mm, ML: +3.60 mm; the atlas of Paxions and Watson) for ECoG recording, and the subcutaneous electrodes was implanted to apply transcranial direct current stimulation to the brain. A M2 brass ring (centered on midline, 2 mm caudal to bregma) and M3 brass ring (centered on midline, 3 mm caudal to lambda) nut was used for epicranial electrode, a conductive paste (Ten20) was applied underneath and inside the epicranial electrodes so that the full surface area (including the hole) of the electrodes was used as stimulation surface. Both anterior and posterior epicranial electrode were connected to 2-pin connector with wire, the connector was subsequently fixed to the skull with dental cement.

**3.2 ECoG signal recording and processing**

**3.2.1 Electrophysiological recordings**

The rats were allowed to recover for about a week following surgery. After implantation, the mice were housed individually in transparent barrels in a sound-proofed recording chamber with insulation and maintained on a 12-h light/12-h dark cycle with lights on at 7:00 AM. They were provided with ad libitum access to food and water. The mice were acclimated to the recording cable for at least 3 days before starting the recording process. The cortical ECoG signals were amplified, filtered and digitalized at a 1000 Hz resolution using a tethered data acquisition system (Medusa, Bio-Signal Technologies, China), and synchronized with video capturing.

**3.2.2 ECoG Data Processing:**

In this study, all ECoG data saved in .edf format were processed using custom scripts written in MATLAB R2023b. Specifically, MATLAB's built-in Butterworth filter was applied to perform low-pass filtering on the data, eliminating high-frequency noise. Additionally, the Welch method was employed to estimate the power spectral density (PSD) for 200-second data segments of each sample. During this process, a Hamming window was used as the analysis window, with a window size of 5000 data points and an overlap of 2500 data points between consecutive windows, ensuring continuity and accuracy in the analysis. For rhythm analysis of ECoG signals, frequency bands were classified as follows: δ band (0.5 - 4 Hz), θ band (4 - 8 Hz), α band (8 - 13 Hz), and β band (13 - 30 Hz).

**3.2.3 θ epoch detection and Closed-loop DCS stimulation**

ECoG signals, sampled at 1000 Hz, were filtered between 0.1 - 200 Hz using a zero phase Butterworth filter of order 4, to derive local field potentials (LFP). Power spectra estimation was performed for 5 min baseline recording by calculated root mean square (RMS) power within the θ frequency band (default 4 - 6 Hz), the value of the mean θ peak was then used in the close-loop setup. Then the RMS power was calculated online and θ epoch was identified as segments of the recorded voltage signal where power in the θ frequency band exceeded three standard deviations above mean θ activity. To enforce strictness in the identification of θ epochs, the minimum duration necessary for detection as a θ epoch was fixed to be 1s.

The closed-loop DCS stimulation was delivered to the animal at the occurrence of θ epoch. The stimulation electrodes were directed to a current stimulator (MCS, Model 4002). A stimulus trigger was generated when a θ epoch was detected, and start to deliver a 250 μA DC stimulation. The stimulation was keep applying with continuously detection of θ epochs, and stopped with the termination for θ epoch. All closed-loop recording and stimulation sessions were performed during the awake state of the rats. The experiments were conducted during the light phase (9:00 AM–5:00 PM). Each daily stimulation session lasted 2 hours. Throughout the stimulation period, the animals were visually monitored to ensure they remained awake and mobile.

**3.2.4 Assessment of mechanical allodynia**

The animals underwent environmental acclimatization and baseline pain sensitivity testing on day 4 before the surgery. Behavioral testing was conducted on all animals on days 7, 8, 9, and 10 following the SNI surgery. For the mechanical withdrawal threshold test, rats were individually placed in a testing chamber consisting of a 30 × 30 × 30 cm plastic box with a transparent plastic floor containing 0.5 cm diameter holes. Each rat was allowed to acclimate to the environment for 30 minutes prior to testing. The withdrawal threshold was determined using the "up-down method" and expressed as the mean withdrawal threshold. Testing began with a filament exerting a force of 2 g, with each filament applied three times at 5-second intervals. If a positive response was observed, the next filament with a lesser force was used; otherwise, the next filament with a greater force was applied. A quick withdrawal or licking of the paw in response to the stimulus was recorded as a positive response. The possible score range was from 0.26 g to 26 g.

**4.** **Cell Viability and Cytotoxicity Assays**

**4.1 Cell proliferation assay**

PC12 cells were seeded in 96-well plates at a density of 1 × 10^4^ cells per well and cultured with different concentrations of TA. After 1 day or 5 days of treatment, 10 µL of CCK-8 solution was added to each well. Cells were incubated for an additional 2 hours, and the absorbance was measured at 450 nm using a microplate reader.

**4.2 Live/dead cell staining**

The cell viability on the 1st or 5th day was assessed using a Live/Dead Viability Kit assay. After PC12 cells were cultured with hydrogels treated with TA solution with different concentrations for 1 or 5 days, 1 μM calcein-AM and 3 μM PI was added and cells were incubated in dark for 30 minutes. Afterwards, the cells were observed using a confocal laser scanning microscope.

**4.3** **In vivo biocompatibility of hydrogels**

Eight 8-week-old male SD rats (about 200 g) were selected from Shanghai Shengchang Biotechnology Co., LTD., Shanghai, China. All animal experiments are conducted in accordance with the protocols approved by the local ethics committee and China's regulations on laboratory animal management. Before implantation in vivo, the prepared hydrogel was purified in PBS (pH = 7.4) for 2 days to remove unreacted monomers. The hydrogel is then soaked in 75% alcohol for 24 hours to disinfect. Finally, the sterilized hydrogel is dipped into the sterilized PBS to remove residual alcohol. Procedure: SD rats were anesthetized with isoflurane and part of the back fur was shaved. Sterilized PFAPT hydrogel (D = 1 cm, H = 1 mm) was implanted into different subcutaneous muscle spaces. Subsequently, the tissue was dewaxed and treated with hematoxylin and eosin (H&E) staining to assess in vivo biocompatibility of hydrogels. Each experimental group consisted of four mice

**4.4 Host immune response to hydrogels and screw electrodes**

Four 8-week-old female SD rats (about 190 g) were selected from Shanghai Shengchang Biotechnology Co., LTD., Shanghai, China. All animal experiments are conducted in accordance with the protocols approved by the local ethics committee and China's regulations on laboratory animal management. Before implantation in vivo, all hydrogels are purified and sterilized according to the previous procedure. During the operation, SD rats were anesthetized with isoflurane. A 5mm craniectomy was performed on the left and right cortices using a portable drill bit. The skull pieces were carefully removed so as not to damage the meninges and cortex. Screw electrodes and PFAPT hydrogel (D = 5 mm, H = 0.5 mm) were then implanted on the surface of the left and right cerebral cortex. Finally, the incision is closed. The rats were placed in heated cages and monitored at 20-minute intervals until the anesthesia had fully recovered. During 4 weeks of implantation, the rats' eating habits and exercise were recorded. After the hydrogel was removed, the brain tissue was collected and then fixed in a 10% neutral buffered formalin solution. After fixation for 24h, the sample was embedded in paraffin wax, followed by tissue dewaxing and staining with GFAP (GB15096, Servicebio, China) and Iba1 (GB153502, Servicebio, China). Fluorescence images were observed under a positive fluorescence microscope (Eclipse C1, Nikon, Japan). DAPI emits blue light at 330 - 380 nm ultraviolet excitation wavelength and 420 nm ultraviolet emission wavelength. FITC emits green light at excitation wavelength of 465 - 495 nm and emission wavelength of 515 - 555 nm. CY3 emits red light at an excitation wavelength of 510 to 560 nm and an emission wavelength of 590 nm. The corresponding fluorescence intensity was calculated using ImageJ software. The average fluorescence intensity was calculated using the surrounding tissue (200 μm).

**4.5 Foreign body reaction (FBR) evaluation**

To assess hydrogel-induced FBR, PFAPT hydrogel was implanted subcutaneously on the back of 9 SD rats (Shanghai Shengchang Biotechnology Co., LTD., Shanghai, China). After 1 and 2 weeks, the surrounding tissue was collected and then fixed in a 10% neutral buffered formalin solution. After fixation for 24 h, the sample was embedded in paraffin. Subsequently, tissue was dewaxed and α-SMA (GB111364, Servicebio, China), collagen I (GB11023-3, Servicebio, China), CD68 (GB11067, Servicebio, China). China), CD3 (GB15004, Servicebio, China) and DAPI (G1012, Servicebio, China) staining. The corresponding fluorescence intensity was calculated using ImageJ software. The average fluorescence intensity was calculated using the surrounding tissue (200 μm).

**4.6 Sequencing Data Alignment, Filtering, and Quantification**

Raw paired-end FASTQ sequences were processed, where Read 1 contained the barcode and UMI sequences, and Read 2 contained cDNA sequences derived from captured transcripts. Barcode sequences extracted from Read 1 were mapped to spot-based array coordinates, allowing for single-base mismatch correction to account for potential sequencing and PCR errors. To ensure data quality, sequences containing two or more undefined bases (N) or exhibiting base quality scores below Phred 10 were discarded. The retained reads were aligned to the human reference genome (GRCh38) using STAR. Alignments achieving a MAPQ score greater than 10 were retained, quantified, and annotated to their corresponding genes. Finally, a spatially resolved expression profile matrix was generated, quantifying the captured transcripts at each spatial coordinate for downstream analyses.

**4.7 Data Reduction, Unsupervised Clustering, and Manual Domain Annotation**

For each spatial transcriptomics sample, dimensionality reduction, unsupervised clustering, and spatial mapping of cellular domains were performed. Briefly, processed gene expression matrices were imported into the Seurat R package (v5.0.3), Gene expression levels were normalized per spot using the NormalizeData function. This involved scaling the Unique Molecular Identifier (UMI) counts by the total counts per spot using a scale factor of 10,000, followed by natural log transformation with a pseudocount of 1. The normalized matrix was subsequently scaled and centered using the ScaleData function. Highly variable genes (HVGs) were identified from this scaled matrix using the FindVariableFeatures function with the "vst" selection method. Principal Component Analysis (PCA) dimensionality reduction was then conducted on the top 2,000 HVGs using the RunPCA function. A shared nearest neighbor (SNN) graph was constructed utilizing the first 50 principal components (PCs). Cell clusters were identified via the Louvain community detection algorithm applied to this SNN graph, grouping spots based on transcriptional similarity. Resulting clusters were manually annotated into six distinct tissue domains based on classical marker genes. Spatial mapping of these annotated domains was visualized using the SpatialDimPlot function.

**4.8 Spatial Cell Type Annotation**

Spatial cell type deconvolution was performed using Cell2location (v0.1.4),This analysis inferred the spatial distribution of the following specific cell types within the tissue: Astrocytes, Entorhinal cells, Macrophages, and cells exhibiting a Neurogenesis signature.

**4.9 Gene Set Expression Scoring within Tissue Domains**

Canonical gene sets representative of specific biological pathways were curated. Gene signature scores quantifying pathway activity were calculated per spot for each curated gene set assigned to corresponding tissue domains using the AddModuleScore function in Seurat.

**5. Statistical Analysis**

In the statistical analysis for comparison between two data groups, the two-sample Student’s t-test was used, and the significance threshold was *P < 0.05.

**Table S1.** Synthesis of PFAPT hydrogels with different contents of PF127-DA, ALGMA and PEDOT:PSS. The contents of LAP was set as 0.2 wt%. The PFAPT hydrogels were expressed as PFS_x_P_y_ according to the content of ALGMA and PEDOT:PSS.


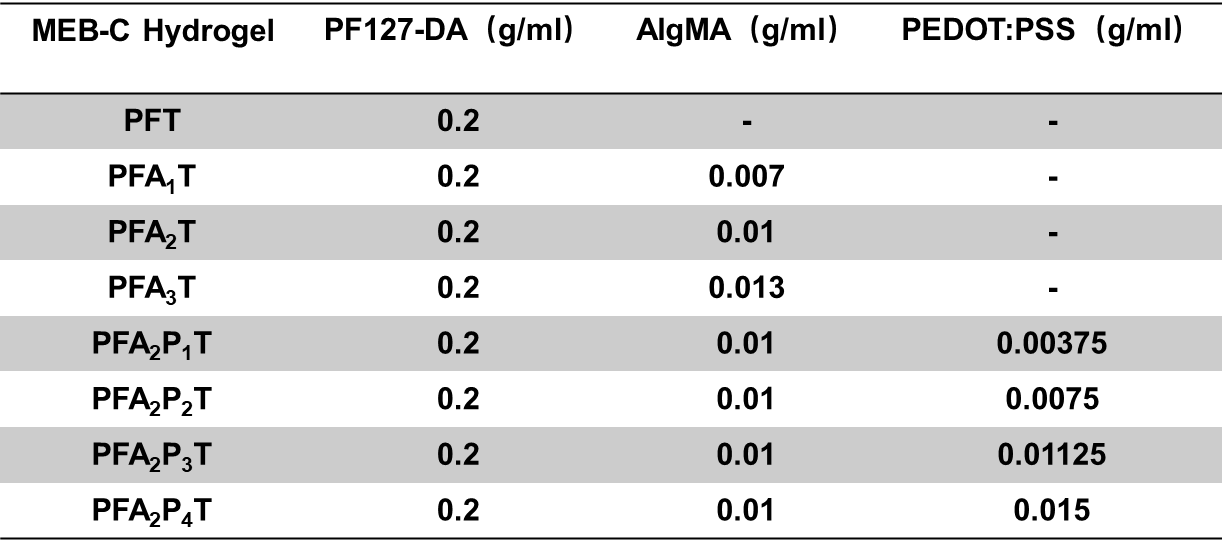


**Table S2.** Clinical information of participant in clinical experiment. Footnote:HC, health control; NP, neuropathic pain; VAS, visual analog scale (0–10); DN4, Douleur Neuropathique 4 questions (0–10); PSQI, Pittsburgh Sleep Quality Index (0–21); PHQ9, Patient Health Questionnaire-9 (0–27); GAD7, Generalized Anxiety Disorder 7-item scale (0–21). Data are presented as mean ± SD or number of participants. t/X² values are from independent t-test or chi-square test; p < 0.05 indicates statistical significance.

|  | | **HC (n=32)** | **NP (n=32)** | **t/X** | **p** |
| --- | --- | --- | --- | --- | --- |
| **Sex（Male/Female）** | | 14/18 | 15/17 | 0.063 | 0.802 |
| **Education years（Mean±SD）** | 0/2/21/9 | 1/5/20/6 | 2.910 | 0.406 |  |
| **Age（Mean±SD）** | | 65.53±8.22 | 66.91±8.65 | -0.673 | 0.506 |
| **VAS（Mean±SD）** | | 0.00±0.00 | 5.72±2.65 | -12.19 | ＜0.001 |
| **DN4（Mean±SD）** | | 0.00±0.00 | 4.72±1.35 | -19.79 | ＜0.001 |
| **PSQI（Mean±SD）** | | 5.25±4.06 | 9.91±4.15 | -4.53 | ＜0.001 |
| **PHQ9（Mean±SD）** | | 2.47±2.60 | 6.78±5.92 | -3.77 | ＜0.001 |
| **GAD7（Mean±SD）** | | 1.84±3.45 | 3.75±4.96 | -1.78 | 0.079 |

**a**

Dry DCM

TEA

**b**

pH=8

Ice bath

**Figure S1.** a) The synthesis of PF127-DA. b) The synthesis of AlgMA.


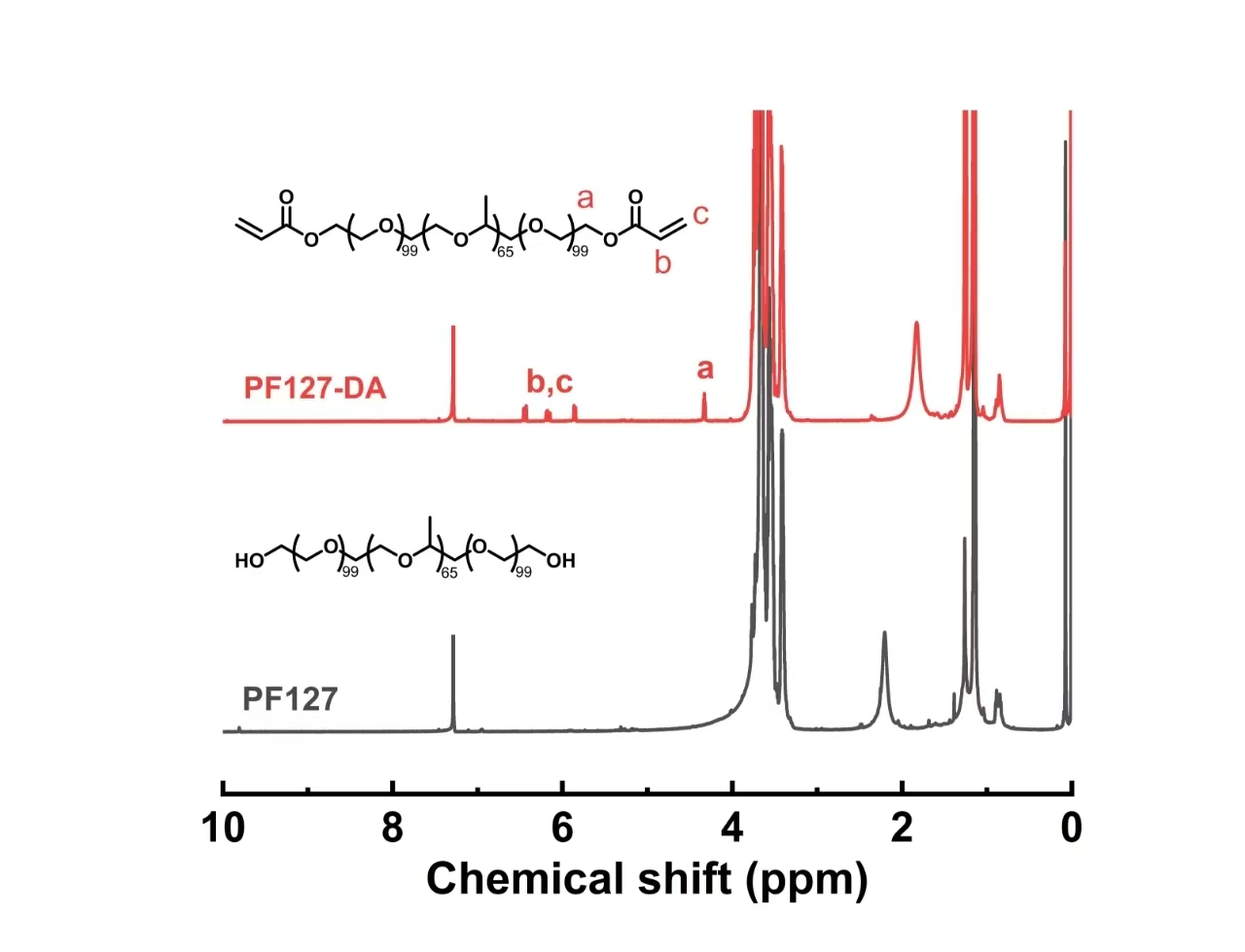


**Figure S2.** ^1^H NMR spectra of PF127, PF127-bis-DA.


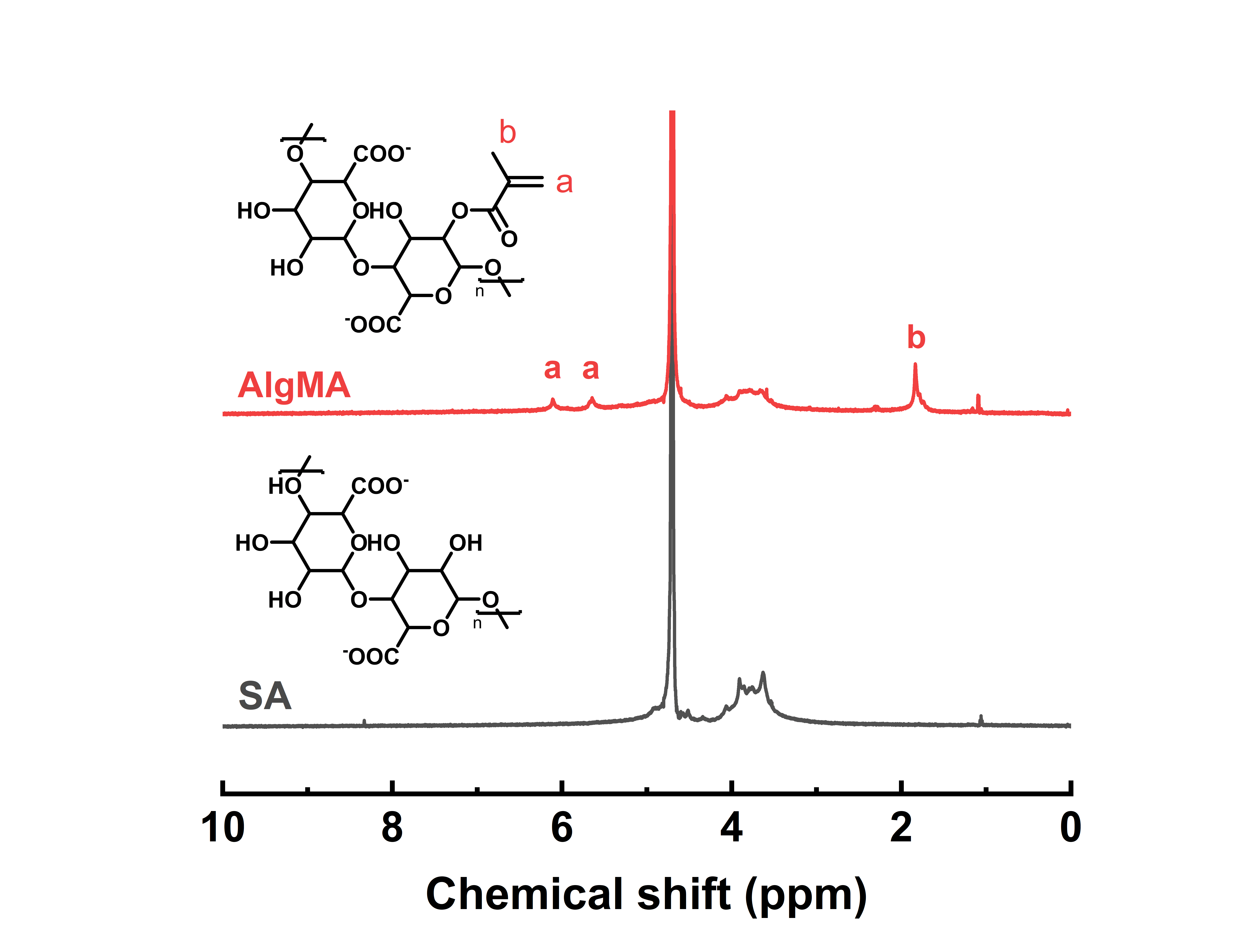


**Figure S3.** ^1^H NMR spectra of SA, AlgMA.


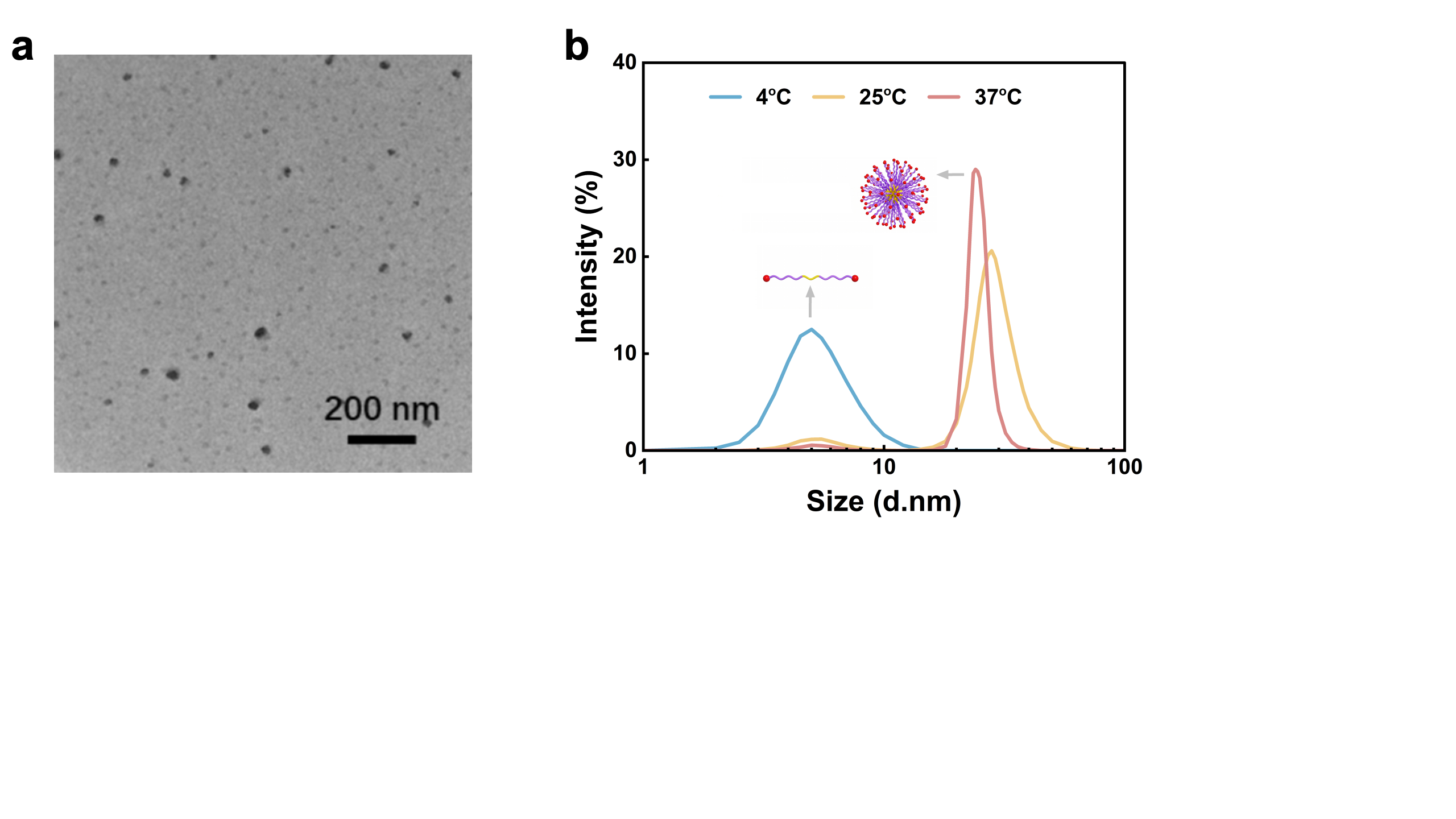


**Figure S4.** (a) TEM image of PF127-DA micelles at room temperature. (b) Temperature-dependent DLS profiles of the PF127-DA at 4, 25, and 37^o^C.

**
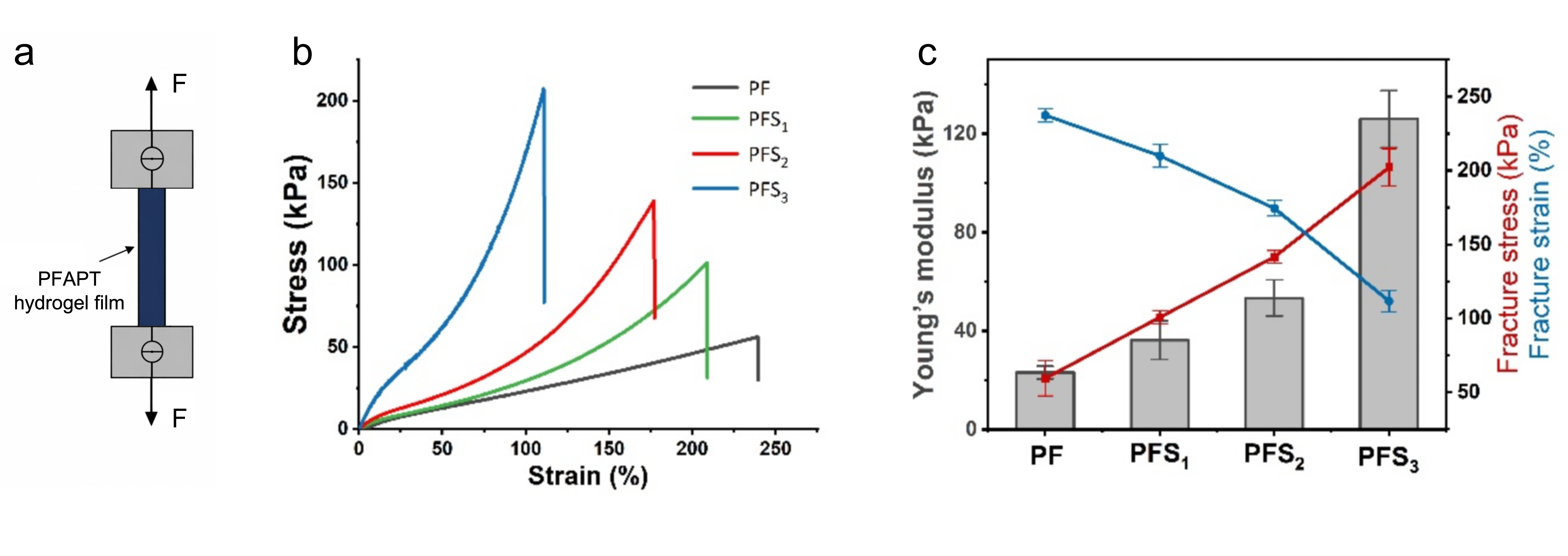
**

**Figure S5.** (a) Schematic illustration of the tensile testing setup for the PFAPT hydrogel. (b) Stress-strain curves. (c) Young’s modulus, fracture stress, and fracture strain of the PFAPT hydrogel with different AlgMA contents.

**Figure S6.** Stress-strain curves of FPS2P2 under cyclic loading-unloading tests at different strains without waiting time between two consecutive loadings (Right). Magnifying stress-strain curves between 0–40% (Left).

**Figure S7.** CV curves for PFA_2_P_2_T hydrogel and bare Pt electrode.


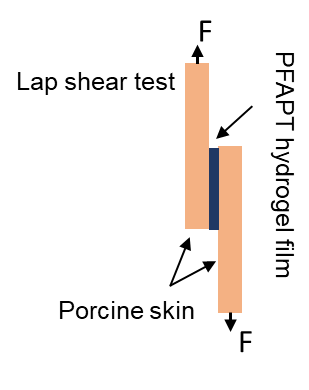


**Figure S8.** Schematic illustration of lap shear tests.

**
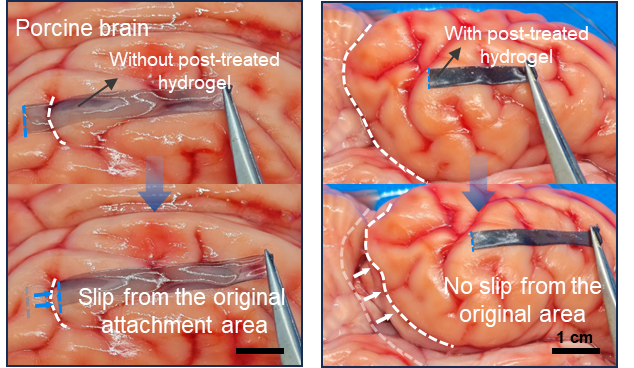
**

**Figure S9.** Comparison of brain tissue integration of the PFAPT hydrogels with or without post-treated.


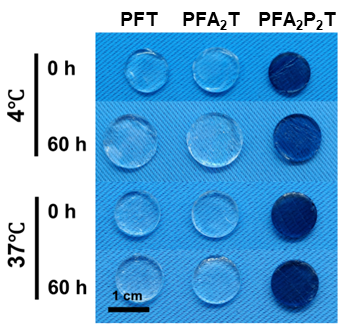


**Figure S10.** The photographs of PFT, PFA_2_T, and PFA2P_2_T gels immersed in standard PBS buffer with different temperatures.


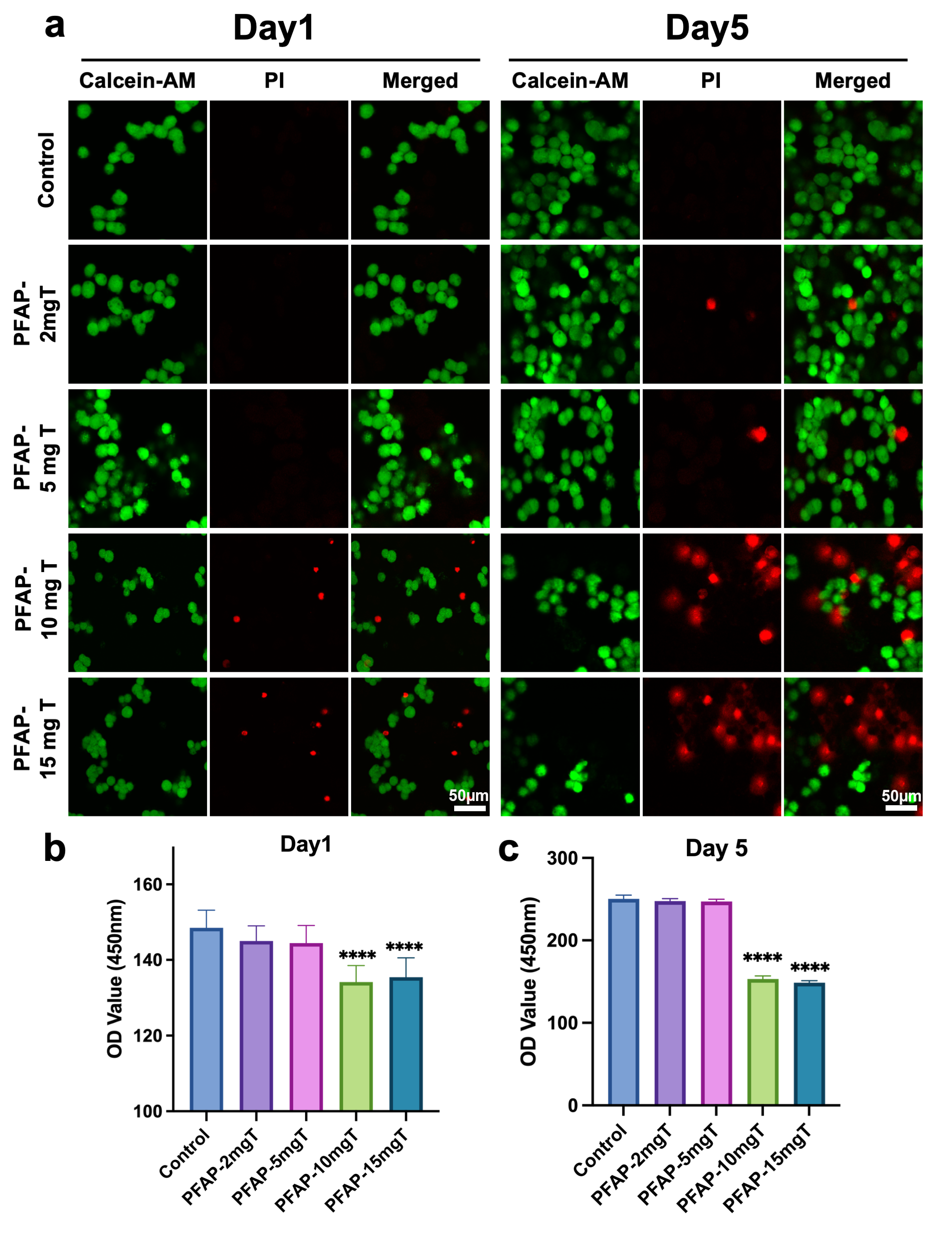


**Figure S11.** The cytocompatibility of PFAPT hydrogels with different TA concentration. (a) Live/dead fluorescent staining of BMSCs cultured on different hydrogels after incubation for 1 and 5 days (red fluorescence indicates dead cells, green fluorescence indicates live cells). (b, c) Proliferation of BMSCs on different hydrogels after incubation for 1 and 5 days.


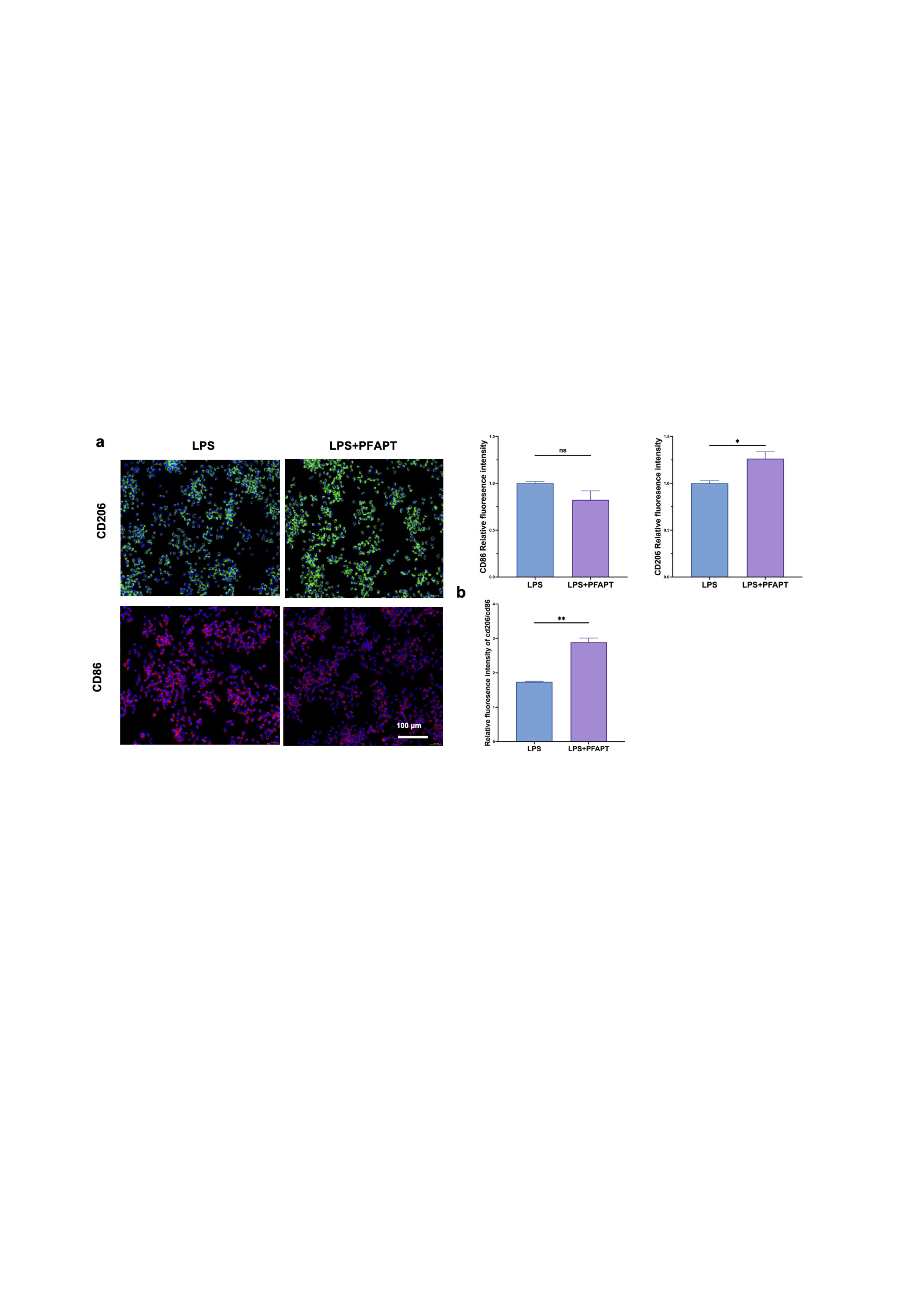


**Figure S12.** (a) Immunofluorescence staining results and quantitative analysis of CD86 (M1) and CD206 (M2) expression in RAW264.7 cells cultured with PFAPT hydrogels. (b) The ratio of CD86 and CD206 of the quantitative analysis.

**
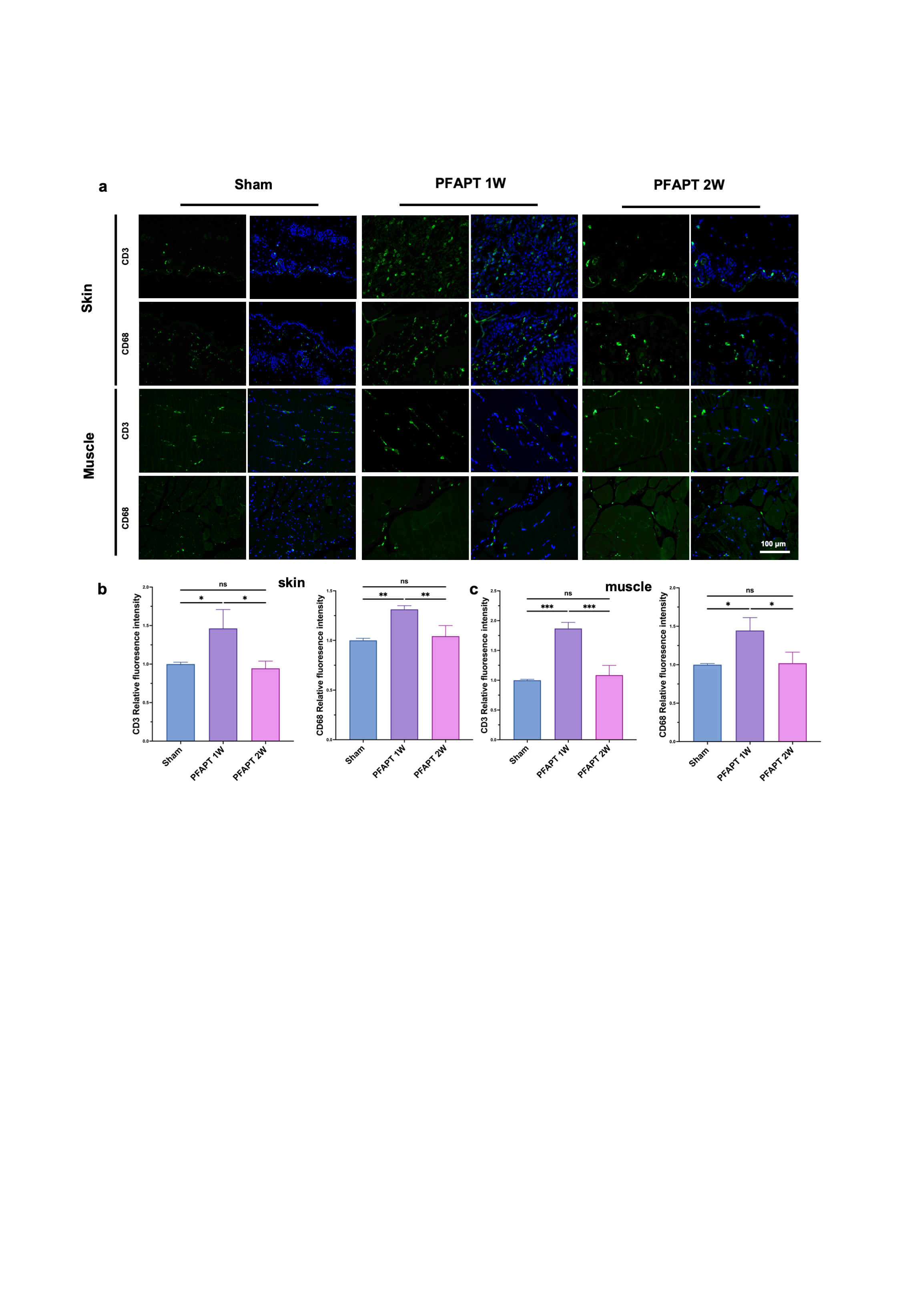
**

**Figure S13.** (a) Immunofluorescent staining of skin and muscle stained with DAPI (nuclei; blue), CD3 (green), and CD68 (green) following in vivo implantation in for 1 week and 2 weeks. (b, c) The corresponding quantitative analysis of CD3 (b) and CD 68 (c).


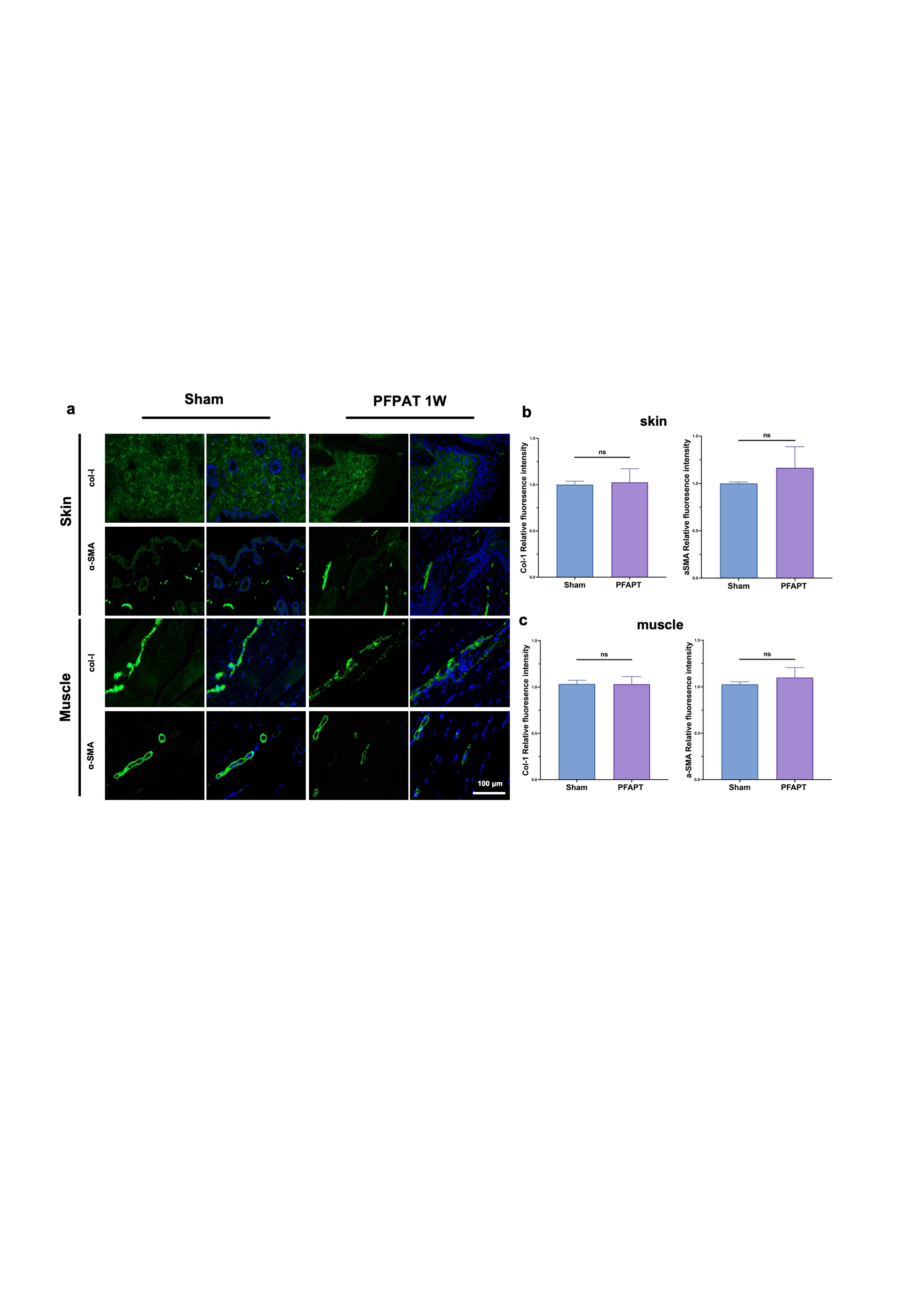


**Figure S14.** (a) Immunofluorescent staining of skin and muscle stained with DAPI (nuclei; blue), col-1 (green), and α-SMA (green) following in vivo implantation in for 1 week. (b, c) The corresponding quantitative analysis of col-1 (b) and α-SMA (c).


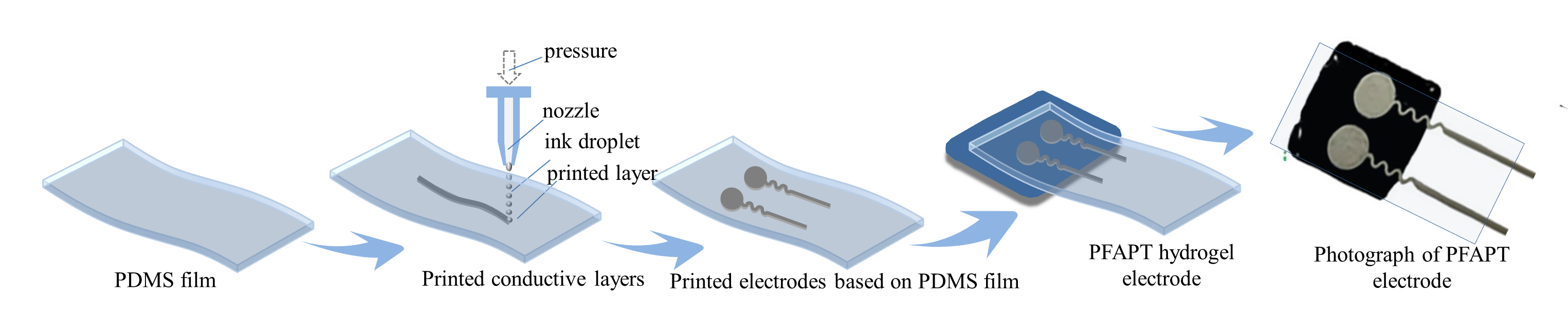


**Figure S15.** The fabrication process of PFAPT hydrogel electrode.


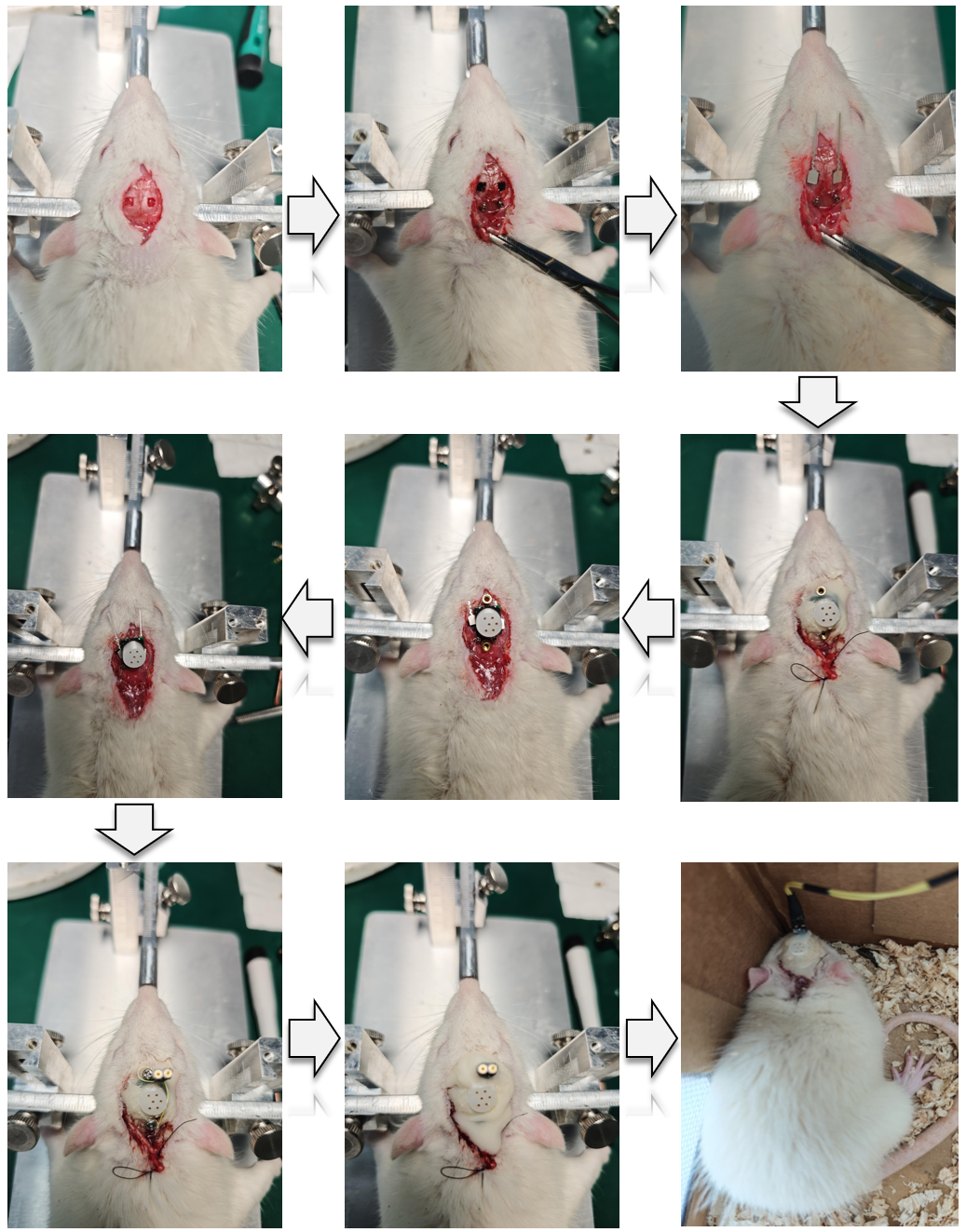


**Figure S16.** Photographs of the electrode implantation process.

**
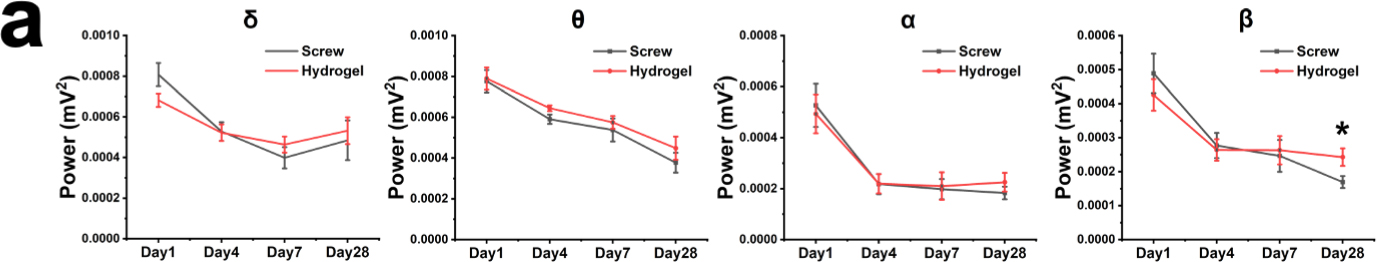
 Figure S17.** From left to right, the energy intensity of δ, θ, α, and β bands in ECoG signals collected on days 1, 4, 7, and 28 post-implantation for hydrogel electrodes and skull screw electrodes is presented.


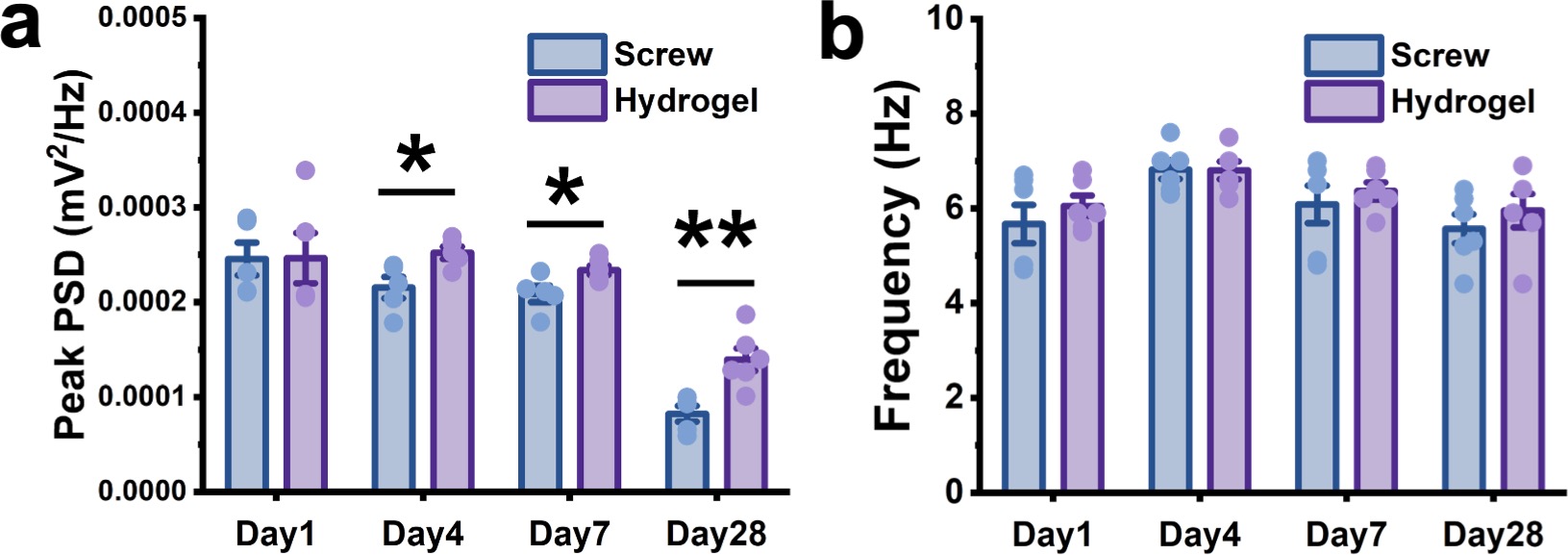


Figure S18. (a) The peak values of the PSD curves in the θ bands for ECoG signals collected on days 1, 4, 7, and 28 post-implantation for hydrogel electrodes and skull screw electrodes. (b) The frequencies corresponding to the peaks. * indicates P < 0.05, and ** indicates P < 0.01.
